# Supplementary material for: Retinoic Acid Is Essential for Th1 Cell Lineage Stability and Prevents Transition to a Th17 Cell Program
Source: Immunity. 2015 Mar 17;42(3):499–511. doi: 10.1016/j.immuni.2015.02.003 (PMC4372260; doi:10.1016/j.immuni.2015.02.003)
Supplement: Document S1. Figures S1–S7, Tables S1–S5, and Supplemental Experimental Procedures [file mmc1.pdf]

Immunity
Supplemental Information

Retinoic Acid Is Essential for Th1 Cell Lineage Stability and Prevents Transition to a Th17 Cell Program

Chrysothemis C. Brown, Daria Esterhazy, Aurelien Sarde, Mariya London, Venu Pullabhatla, Ines Osma-Garcia, Raya al-Bader, Carla Ortiz, Raul Elgueta, Matthew Arno, Emanuele de Rinaldis, Daniel Mucida, Graham M. Lord, and Randolph J. Noelle

Figure S1 (related to Figure 1). Expression of Foxp3 in CD4$^+$ T-cells deficient in RA signaling

(A) Intracellular expression of Foxp3 in CD4$^+$ T-cells from spleen, thymus and mesenteric lymph nodes (MLN) of wild-type littermate control (WT) and dn*Rara* mice.

(B) Total number of CD4$^+$Foxp3$^+$ T-cells in spleen (upper panel) and thymus (lower panel) of WT and dn*Rara* mice

Data are representative of two independent experiments. Mean ± SEM.

Figure S1.

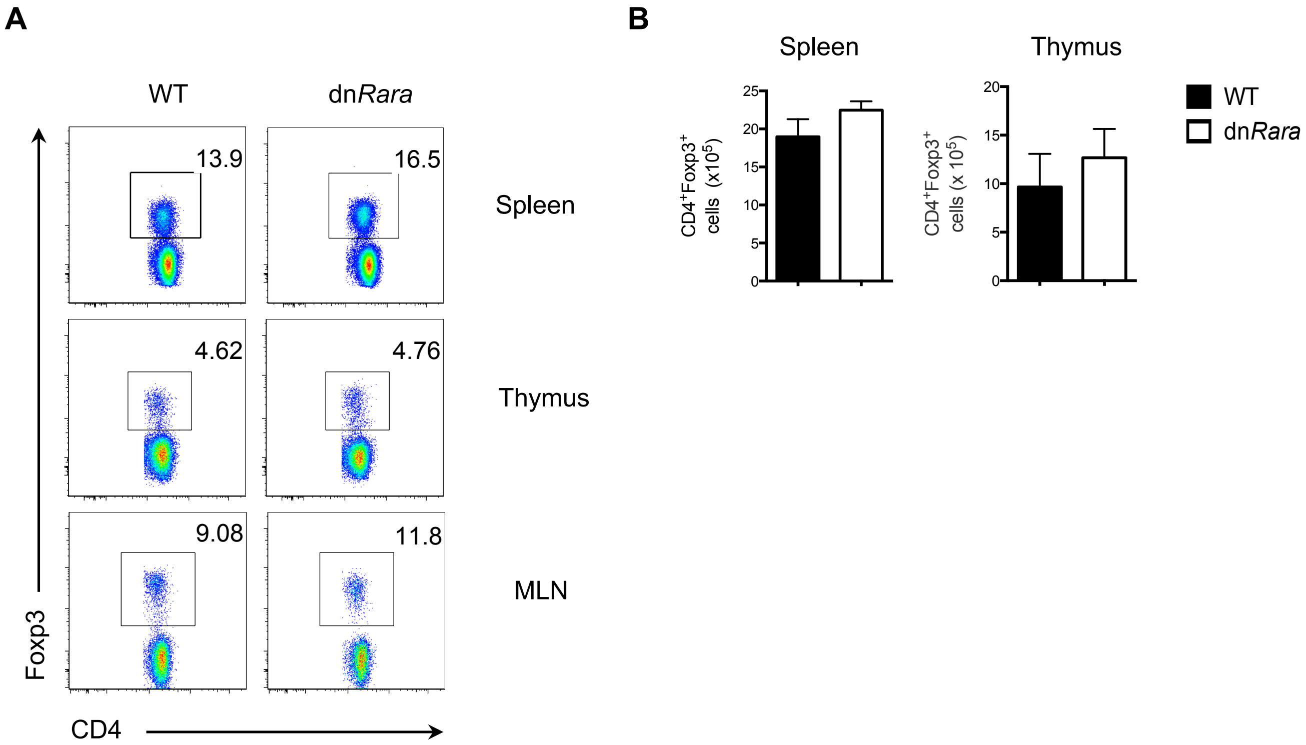
A
B
WT
dnRara
13.9
16.5
Spleen
4.62
4.76
Thymus
9.08
11.8
MLN
Foxp3
CD4
Spleen
Thymus
CD4+Foxp3+ cells (x10^5)
25
20
15
10
5
0
CD4+Foxp3+ cells (x 10^5)
20
15
10
5
0
WT
dnRara

Figure S2 (related to Figure 2). Proliferation and differentiation of CD4$^+$ T-cells in the absence of RA signaling

(A) Naïve CD4+ T-cells from WT and dn*Rara* mice were labeled with CellTrace^{TM} and cultured under Th1 conditions for 5 days. Flow cytometry showing dye dilution, gated on viable CD4$^+$ T-cells.

(B) Cell-surface expression of CD44 and CD25 on naïve CD4+ T-cells from WT or dn*Rara* mice cultured under Th1 conditions for 5 days.

(C) Naïve CD4+ T-cell from WT and dn*Rara* mice were cultured under Th0 or Th2 conditions for 6 days. Cells were analysed by flow cytometry for expression of intracellular RORγt. Gated on CD4$^+$ T-cells.

(D) Sorted naïve CD4$^+$ T- cells from WT and dn*Rara* mice were cultured under Th17 conditions for 6 days. Intracellular IL-17A and IFN-γ expression after stimulation with PMA and ionomycin.

(E) CD4$^+$ T-cells from dn*Rara-Ifng*^{eYFP} and *Ifng*^{eYFP} mice were cultured under Th1 conditions. Quantitative real-time PCR analysis of *Cxcr3* and *Il12rb2* from IFN-γ^+ (eYFP$^+$) cells sorted on day 7. Samples from three independent experiments.

Representative data from two to three independent experiments (A-D). Mean ± SEM.

Figure S2.

A

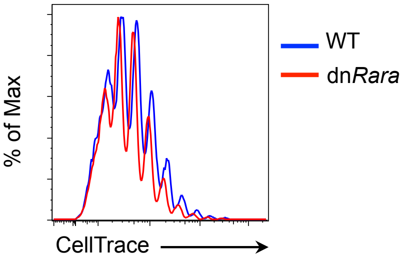

	WT	dn*Rara*
% cells divided	90.83	92.67
Proliferation index	3.65	3.86

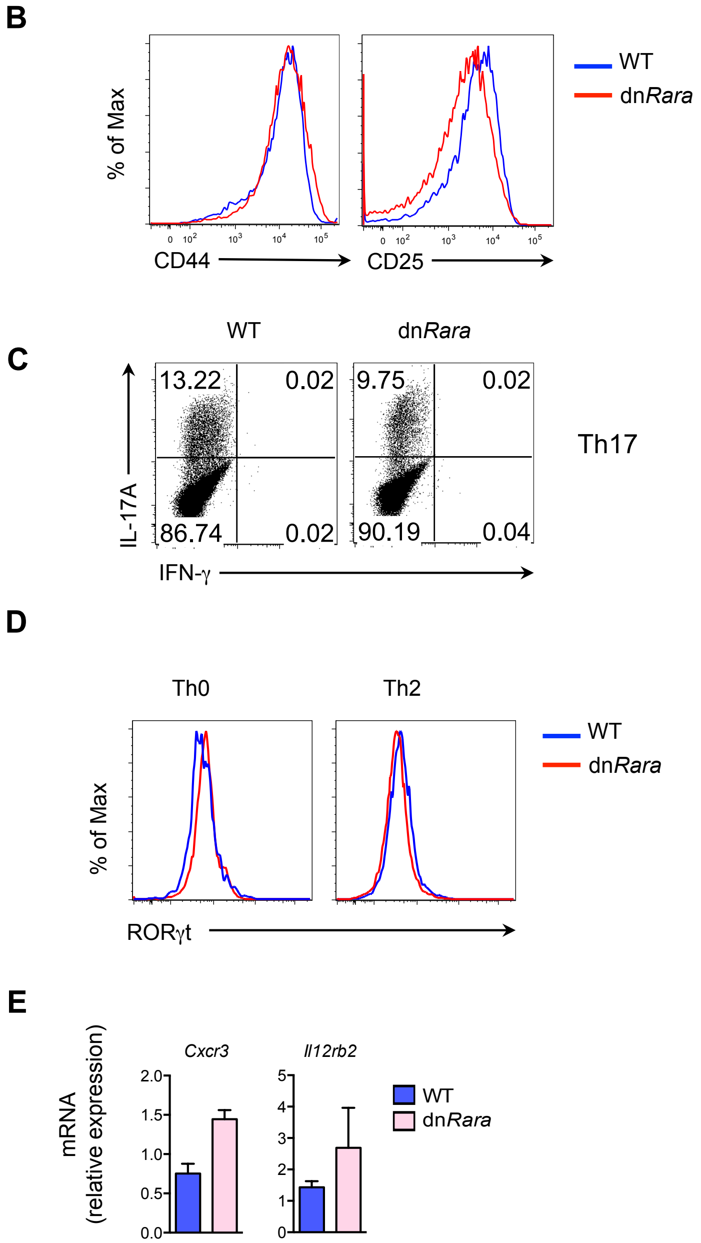

Figure S3 (related to Figure 3). STAT3 and STAT4 activity in dn*Rar*a Th1 differentiated cells

(A) Flow cytometric analysis of STAT3 and STAT4 phosphorylation in naïve $CD4^+$ T-cells from dn*Rara* and WT mice differentiated under Th1 conditions. Cells analysed after 6 days following treatment with 25ng/ml IL-12, 20ng/ml IL-6 and 10ng/ml IL-23 for 30 minutes. Dashed lines represent untreated cells.

(B) Bar graph depicts ratio of pSTAT3/pSTAT4 signaling as assessed by MFI.

Figure S3.

A

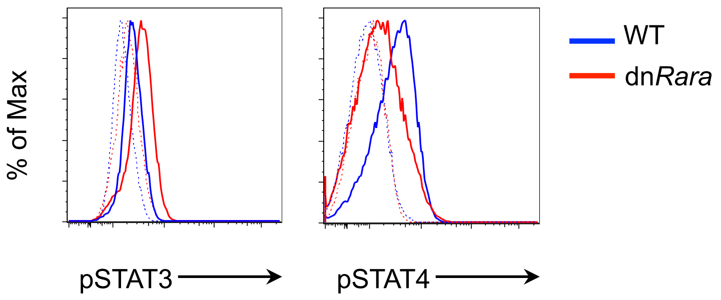

% of Max
pSTAT3
pSTAT4
WT
dn*Rara*

B

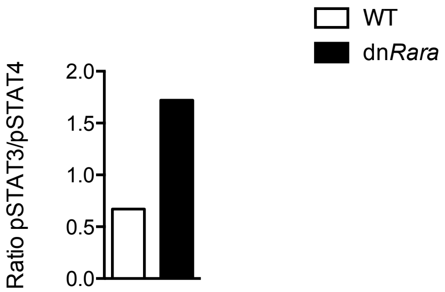

WT
dn*Rara*
Ratio pSTAT3/pSTAT4
2.0
1.5
1.0
0.5
0.0

Figure S4 (related to Figure 4). Cytokine analysis following temporal inhibition of RA signaling in Th1 cells

(A) Naive $CD4^+$ T-cells from dn$Rara^{lsl/lsl}$ mice were cultured under Th1 conditions. Th1 cells were transduced with TAT-Cre on days 5 and 7 and repolarised under Th1 conditions for a further 5 days. Intracellular expression of IFN-γ and IL-17A following PMA and ionomycin stimulation.

(B) Naive CD4+ T-cells from $Ifng^{eYFP}$ mice were differentiated under Th1 conditions. IFN-γ^+ ($eYFP^+$) cells were sorted on day 7 and recovered cells underwent secondary repolarisation in Th1 conditions for 5 days in the presence of Veh or RAi. Intracellular expression of IFN-γ and IL-17A following PMA and ionomycin stimulation.

Data representative of two independent experiments.

Figure S4.

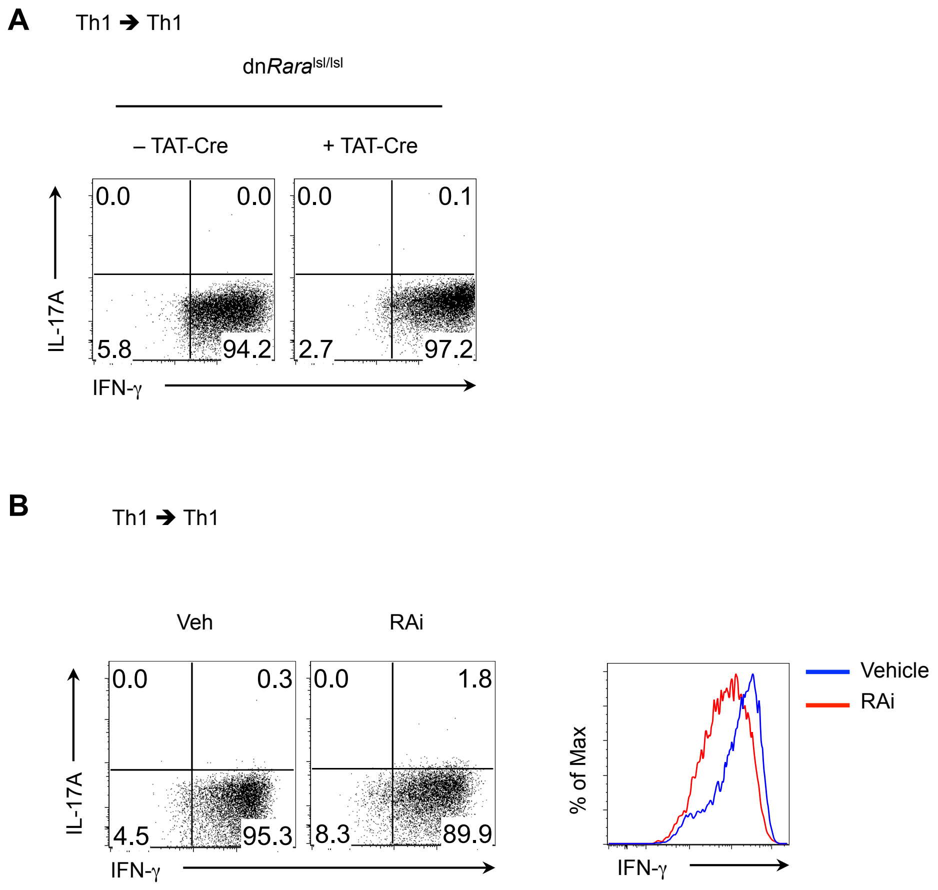

A
Th1 ➔ Th1
dn*Rara*lsl/lsl
– TAT-Cre
+ TAT-Cre
0.0
0.0
5.8
94.2
0.0
0.1
2.7
97.2
IL-17A
IFN-γ
B
Th1 ➔ Th1
Veh
RAi
0.0
0.3
4.5
95.3
0.0
1.8
8.3
89.9
IL-17A
IFN-γ
% of Max
IFN-γ
Vehicle
RAi

Figure S5 (related to Figure 5). RA-RARα regulates enhancers at Th1 genes and represses Th17 lineage specifying genes

Naive $CD4^+$ T-cells from dn*Rara* and WT mice were cultured under Th1 conditions as in Figure 5. After 6 days, ChIP was performed with the specified antibodies, followed by real-time PCR analysis at selected sites (B-C) or sequencing (A).

(A) ChIP-seq binding tracks at *Stat4* and *Ifng* loci for RARα in WT Th1 polarised cells and p300 binding, H3K27ac, H3K4me1 and H3K4me3 modifications in WT and dn*Rara* Th1 cells.

(B) Validation of the RARα ChIP-seq regions in (A) by ChIP-qPCR assays. Untr6 region serves as a negative control. Data presented normalised to input.

(C) ChIP analysis of the abundance of p300 at the loci in (B) in WT and dn*Rara* Th1 cells. Data presented normalised to input.

(D) ChIP-seq analysis of STAT4 binding at the Tbx21 enhancer and comparison of p300 binding in WT and $STAT4^{-/-}$ Th1 cells. ChIP-Seq data (Vahedi et al. 2012 and Wei et al., 2010) was mapped to the Dec. 2011 (GRCm38/mm10) mouse genome assembly with the UCSC genome browser along with the ChIP-seq binding track for RARα at the *Tbx21* locus.

(E) Quantitative real time PCR analysis of selected genes identified as differentially expressed on genome wide transcriptional profiling analysis of cells as in (A). Mean ± SEM.

(F) Cell-surface expression of IL6-Rα by flow cytometry in naïve dn*Rara* and WT $CD4^+$ T-cells at indicated timepoints. Grey histogram indicates staining for isotype control.

Data (B-F) representative of two to three independent experiments. Mean ± SD unless otherwise stated, $^{**}p < 0.01$; $^{****}p < 0.0001$.

Figure S5.

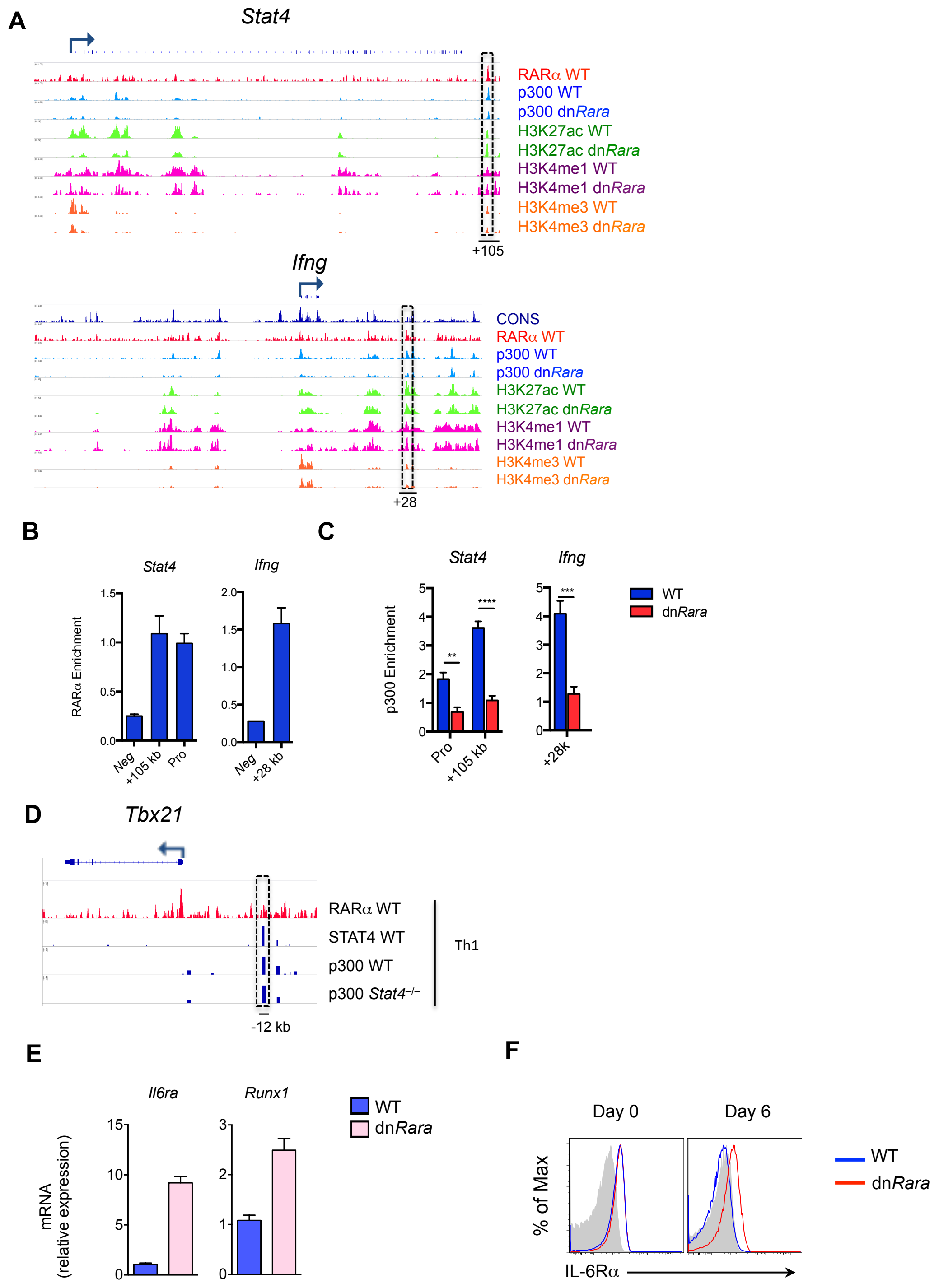
A
Stat4
RARα WT
p300 WT
p300 dnRara
H3K27ac WT
H3K27ac dnRara
H3K4me1 WT
H3K4me1 dnRara
H3K4me3 WT
H3K4me3 dnRara
+105
Ifng
CONS
RARα WT
p300 WT
p300 dnRara
H3K27ac WT
H3K27ac dnRara
H3K4me1 WT
H3K4me1 dnRara
H3K4me3 WT
H3K4me3 dnRara
+28
B
Stat4
Ifng
RARα Enrichment
Neg
+105 kb
Pro
Neg
+28 kb
C
Stat4
Ifng
p300 Enrichment
WT
dnRara
Pro
+105 kb
+28k
D
Tbx21
RARα WT
STAT4 WT
p300 WT
p300 Stat4–/–
Th1
-12 kb
E
Il6ra
Runx1
mRNA (relative expression)
WT
dnRara
F
Day 0
Day 6
% of Max
IL-6Rα
WT
dnRara

Figure S6 (related to Figure 6). Cytokine production by dnRARa T-cells following infection with L. monocytogenes

(A) Splenocytes from dn*Rara* and WT mice infected with Lm-2W were restimulated with LLOp for 24 h. Concentration of IFN-γ, IL-17A and IL-4 in supernatants was measured by multiplex bead array (Biorad). Data normalised to total numbers of $CD4^+$ T-cells. n = 3-4 mice per group.

(B) Intracellular staining for IFN-γ and IL-4 following stimulation of splenocytes with LLOp for 6 h, 7 days after infection with *L. monocytogenes*. Gated on $CD3^+CD4^+$ T-cells

(C) Cell surface expression of IL-6Rα by flow cytometry on LLOp:I-A^b CD4+ T-cells isolated from spleen of dn*Rara* or WT mice 7 days after infection with *L. monocytogenes*. Data from 4 pooled mice. Numbers indicate MFI.

Data representative of two to three independent experiments. Mean ± SEM.

Figure S6.

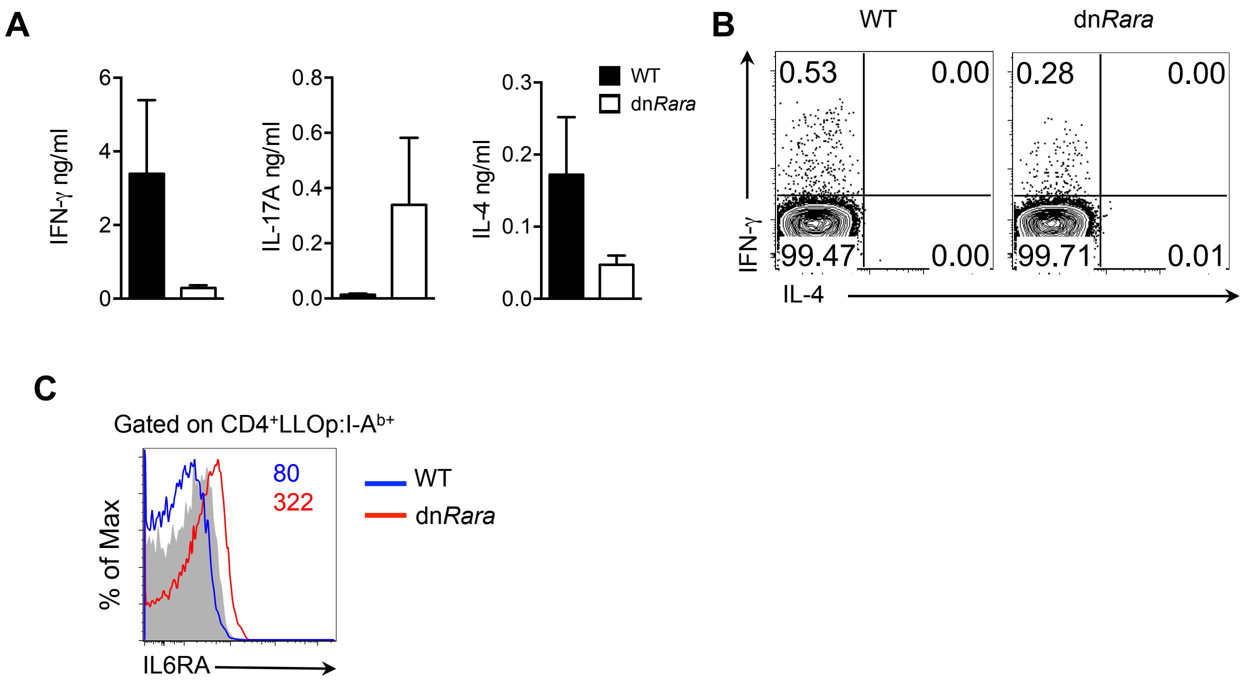
A
IFN-γ ng/ml
IL-17A ng/ml
IL-4 ng/ml
WT
dn*Rara*
B
WT
dn*Rara*
0.53
0.00
99.47
0.00
0.28
0.00
99.71
0.01
IFN-γ
IL-4
C
Gated on CD4+LLOp:I-Ab+
80
322
WT
dn*Rara*
% of Max
IL6RA

Figure S7 (related to Figure 7). Gut homing in dn*Rara*-OTII CD4+ T-cells
(A) Percentage of OTII or OTII(dn*Rara*) $CD4^+$ cells recovered from LPL, IEL, MLN and Spleen of $RAG^{-/-}$ recipients, 9 days after adoptive transfer (n = 3-4 per group). Data representative of two independent experiments. Mean ± SEM.

Figure S7.

A

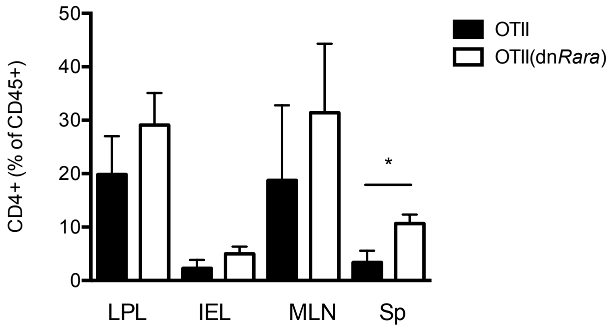

Table S1 (related to Figure 5). List of Sequencing-Based Data Used in This Study including publically available data as indicated by Geo Accession Number

Samples	Non-redundant tags	Peak counts
RARA_WT	13303876	1776
H3K4me1_DNRAR	18605274	65960
H3K4me1_WT	23760603	49542
H3K4me3_DNRAR	18333386	49505
H3K4me3_WT	21918629	53135
H3K27Ac_DNRAR	17421600	37788
H3K27Ac_WT	20513640	37151
H3K27me3_DNRAR	30667883	56002
H3K27me3_WT	20833021	78511
p300_DNRAR	23023765	30495
p300_WT	25213927	46191
Stat4 WTTh1 (GSM550303)	8982352	20862
p300 WT Th1 (GSM994508)	19652779	25554
p300 Stat4$^{-/-}$ Th1 (GSM994509)	18282554	29208

Table S2 (related to Figure 5). Genes downregulated in dn*Rara* Th1 cells that were bound by RARα in WT Th1 cells.

1110037F02Rik	Fli1	Ncln
1810011H11Rik	Fmnl3	Nedd4l
3300005D01Rik	Foxo3	Nfic
5830416P10Rik	Foxp1	Nln
Acsl4	Furin	Nme1
Adora2a	Gas5	Nod1
Alkbh7	Gcsh	Notch2
Asb2	Gfi1	Nt5e
Birc5	Gimap3	P2rx7
Blm	Gimap4	Pde2a
Bre	Gimap8	Prr5l
Capzb	Gimap9	Rbks
Chsy1	Hic1	Rcbtb2
Cmas	Hmgcs1	Shf
Cnga1	Idi1	Slc16a6
Coq7	Ifngr1	Smad3
Ctps	Ifrd2	Sqle
Cycs	Irf8	Sulf2

Cyp51	Itih5	Tbx21
Cyp51	Kcnn4	Treml2
Dennd4a	Kif2c	Txn2
Dusp6	Lbr	Ube2e3
E2f3	Lef1	Uchl3
Enpp4	Mdc1	Vav3
Fasn	Me2	Vipr1
Fgl2	Mrto4	

Table S3 (related to Figure 5). Genes upregulated in dn*Rara* Th1 cells that were bound by RARα in WT Th1 cells

1110038F14Rik	Ifngr2	Slfn2
Ak2	Il15ra	Socs1
Antxr2	Insr	Sp100
Aph1b	Irf1	Stat1
Arhgap25	Irgm1	Tagap
Arid4a	Kif3b	Tmem50a
B2m	Mcl1	Tnip1
Bace2	Mettl8	Tor1aip2
Bcl10	Mga	Traf1
Bcl6	Mpeg1	Trpm6
Birc3	Nek6	Twsg1
Cd320	Net1	Usp53
Cnnm2	Npc2	Vav1
Ddit3	Plec	Wdsub1
Egr2	Polg	Zbp1
Fam43a	Ptpn1	Zfp207
Filip1l	Rab19	Zfp36l2
Fndc3a	Rhd	Zmym6
Fuca1	Slamf1	

Table S4. Taqman assays used for RT-PCR gene expression analyses (related to Figures 1-3 and 5).

Mouse ACTB	4352341E
Il6ra	Mm00439653_m1
Il22	Mm00444241_m1
Runx1	Mm01213404_m1
Batf	Mm00479410_m1
Cxcr3	Mm99999054_s1

Il23r	Mm00519943_m1
Il1r1	Mm00434237_m1
Il21	Mm00517640_m1
Il10	Mm00439616_m1
Irf8	Mm00492567_m1
Irf4	Mm00516431_m1
Stat4	Mm00448890_m1
Il12rb2	Mm00434200_m1
Ifng	Mm00801778_m1
Il12rb1	Mm00434189_m1
Rorc	Mm01261022_m1
Gata3	Mm00484683_m1
Tbx21	Mm00450960_m1

Table S5 (related to Figure 5). Sequences of PCR primers used in ChIP assays

Stat4_+105k F	TCCTCCTCCCTTTGTTGTTC
Stat4 +105k R	GGGCCTTAATCAACCATTTC
Stat4 Promoter F	AGAGGGCATACACCGAGAAC
Stat4 Promoter R	TCTAGGGAGCCAGCATCAAC
Tbx21 Promoter F	TCGCTTTTGGTGAGGACTG
Tbx21 Promoter R	GGTGGCAGGTTGACTCTTTC
Tbx21 -12k F	GCGGAAGAGGGAACTAACAC
Tbx21 -12k R	GGACCCGGAACCTATGTATG
Irf8 Promoter F	CAGAAGCTAGGGCTGGTGTC
Irf8 Promoter R	CACAGAACAGATCCCAAATGTC
Irf8 -11k F	CCTTAACCCCGGAACTGTAG
Irf8 -11k R	TGCTGTGCTTGCCTCTACTC
Il6ra Promoter F	TCCGCTTGAGTTTTGCTTTC
Il6ra Promoter R	CACTGACCTGCCTTCTACTTTAAC
Il6ra +32k F	CAAAGCTAAAACCAGGAAATGAC
Il6ra +32k R	AAAAGGTTCCATGTGATGTTG
Rorc Promoter (Rorγt isoform) F	AGGAATTTGGGTGTGGTGAG
Rorc Promoter (Rorγt isoform) R	CTGTCTTGGGTGGTGTCTTG
Runx1 Promoter 1 F	TGGAAGAGGAAGAAGCTGTG
Runx1 Promoter 1 R	CAAGAGAAGCCACCCCAAAC
Runx1 Promoter 2 F	TGCTGGGCTTACACTTCTGAC
Runx1 Promoter 2 R	TGGACCTCATAAACAACCACAG
IFNg +28k F	CTTTGAGCCACTGATGGGTAG
IFNg +28k R	GCCTCTCCACGTCTCTTCTTC

Supplemental Experimental Procedures

Reagents

$LLO_{190\text{-}201}$ was synthesised by PiProteomics and was >95% pure, as determined by HPLC. LLO:I-A^b monomers were provided by NIH Core Tetramer Facility. PE labeled LLO:I-A^b dextramers were synthesised by Immudex. Recombinant Lm-2W strain was provided by Marc Jenkin's Laboratory. LE540 was purchased from Alpha Laboratories.

Naïve CD4+ T-cell isolation and culture

Naïve $CD4^{+}CD25^{neg}CD44^{lo}CD62L^{hi}$ T-cells were isolated by cell sorting by FACSAria (BD) after enrichment with a $CD4^{+}$ T-cell negative selection kit (Miltenyi Biotec). T-cell depleted splenocytes were prepared using a $CD3^{+}$ microbead selection kit (Miltenyi Biotec) followed by irradiation at 3000 rad. Naïve CD4+ T-cells were cultured for 3 days with irradiated T cell-depleted splenocytes at a ratio of 1:5 in the presence of 5 μg/ml of anti-CD3 (145-2C11) under Th0 cell conditions (IL-2 100 IU/ml, anti-IL-4 (11B11) and anti-IFN-g (XMG1.2), 10 μg/ml each); Th1 cell conditions (100 IU/ml of IL-2, 10 ng/ml of IL-12, and anti-IL-4); Th2 cell conditions (100 IU/ml of IL-2, 10 ng/ml of IL-4, anti-IL-12 (C17.8), and anti-IFN-γ (XMG1.2); or Th17 cell conditions, 5 ng/ml TGFβ, 20 ng/ml IL-6, 10 ng/ml IL-1β, anti-IL-4, and anti-IFN-γ). Cells were expanded for an additional 3-4 days. Where indicated, 10 ng/ml IFN-γ or 10 μg/ml anti-IFN-γ was added. In secondary repolarisation assays, where specified, LE540 (1 μM) or DMSO (vehicle control) was added to the media. Cytokines were from R&D. Anti-CD3 was from BioXcell and other antibodies were from BD Biosciences. All cell cultures were performed in complete RPMI containing 10% fetal

bovine serum (FBS), 55 μM β-mercaptoethanol, HEPES, non-essential amino acids, glutamine, penicillin and streptomycin.

TAT-Cre transduction

Sort purified naïve CD4+ T-cells were differentiated under Th1 conditions. After 5 days, cells were washed twice in serum free medium prior to treatment with 50 μg/ml TAT-Cre (Millipore) or medium alone (mock treatment). Cells were incubated at 37°C for 45 minutes. The reaction was quenched with medium containing 20% FBS followed by further washing. Cells were expanded for 2 days followed by retreatment with TAT-Cre or media as before. Cells were then restimulated under Th1 cell conditions for 3 days and expanded for a further 2 days prior to analysis.

Flow Cytometry

For analysis of cytokine production, cells were restimulated with 100 ng/ml phorbol 12-myristate 13-acetate (PMA) and 500 ng/ml ionomycin in the presence of monensin for 4-5 h at 37°C in a tissue culture incubator. Cell surface staining was carried out in PBS with 2% FBS. For live cell analysis or cell sorting, dead cells were excluded by staining with SYTOX blue (Invitrogen). For intracellular staining, cells were first stained with LIVE/DEAD Fixable Violet or near IR Dead Cell Stain (Invitrogen), followed by staining for cell-surface markers and then resuspended in fixation/permeabilisation solution (Cytofix/Cytoperm kit or Transcription Factor Buffer kit; BD Bioscences). Intracellular staining carried out in accordance with the manufacturer's instructions. Intracellular phosphorylated STAT proteins were stained with Phosflow Lyse/Fix Buffer, and Phosflow Perm Buffer III (BD

Biosciences) according to the manufacturer's protocol. Data were collected with a LSR Fortessa (BD) and results were analyzed with FlowJo software (Tree Star). All the antibodies for staining cell surface markers, cytokines or transcription factors were purchased from either BD Biosciences or eBiosciences.

Luminex Immunoassays

Cytokine levels in supernatants were measured using a multiplex bead-based assay (Bio-Rad Laboratories) in a Luminex FlexMap3D System (Luminex Corporation).

Western Blotting

Differentiated Th1 cells were lysed in RIPA buffer supplemented with protease inhibitors. Lysates were electrophoresed on 10% gels (Biorad), transferred to nitrocellulose and blotted with anti-STAT4 or anti-actin followed by anti-rabbit-horseradish peroxidase conjugated antibody. All antibodies were from Cell Signaling Technology.

***L. monocytogenes* infection and analysis**

Mice were infected i.v. with 1 x 10^6 cfu L. monocytogenes and spleens were harvested 7 days later. For FACS analysis, single cell suspensions were enriched for $CD4^+$ T-cells with a $CD4^+$ T-cell negative selection microbead kit (Miltenyi Biotec) and stained with PE labeled, LLO:I-A^b dextramer (Immudex) and cell surface antibodies. For analysis of cytokine production, supernatants were collected from splenocytes restimulated with LLO peptide (PiProteomics) at 10 μg/ml for 24 h or intracellular cytokine staining was performed following stimulation with LLO peptide for 6 h in the presence of monensin.

Food antigen induced diarrhoea model

Rag1$^{-/-}$ mice were kept on a sulfatrim-containing diet and only exposed to autoclaved supplies. Naïve OTII CD4 cells (defined as $CD4^{+}CD25^{-}Vb5^{+}Va2^{+}CD44^{-}$) were sorted from 8-12 weeks old female C57Bl6 OTII(dn*Rara*) or C57Bl6 OTII mice using a FACS Aria cell sorter (Becton Dickinson), and 2 x 10^{6} cells in 100µl PBS were retro-orbitally transferred to 12 weeks old Rag1$^{-/-}$ females. 12h after the adoptive transfer, the drinking water was replaced by a 1% Grade II ovalbumin (OVA, Sigma) and 0.5% Splenda (McNeil Nutritionals) solution for 7 days. Body weight was measured at 5pm every day. For monitoring diarrhea development, the faeces texture after 7 days of OVA, 2h after a gavage challenge with 50mg Grade III OVA (Sigma) in 200 µl PBS on days 9 and 10 and without further challenge on day 12 was analysed. A mouse was diagnosed with diarrhoea if the faeces had the characteristic soft and light appearance at two consecutive occasions. For the single gavage challenge experiment, mice were subjected to the challenge on day 9 only and the faeces were analysed after 2h. To determine T cell frequencies, lymphocytes were isolated as previously described (Mucida et al., 2007) on day 7 (from mesenteric lymph node (MLN) and spleen only) or day 9 (from the intestinal epithelium, lamina propria, MLN and spleen) after the start of oral OVA exposure of the recipient mice. For cytokine staining, isolated lymphocytes were stimulated for 3h in RPMI medium supplemented with 10% FBS, 55 µM β-mercaptoethanol, 100ng/ml PMA (Sigma), 500ng/ml Ionomycin (Sigma) and 10µg/ml brefeldin A (Sigma) prior to the incubation with antibodies. Cells were first stained with antibodies against cell surface markers, followed by permeabilization using either Fix/Perm buffer (BD Pharmingen) for cytokine stainings, or

using the Foxp3 Mouse Regulatory T cell Staining Kit (eBioscience) for Foxp3 staining. The fluorescent-dye- conjugated antibodies used were obtained from BD-Pharmingen (anti-CD4, 550954; anti-CD25, 553866; anti-IL-17a, 559502; anti-Vb5, 553190) or eBioscience (anti-CD44, 56-0441; anti-CD45.2, 47-0454; anti-TCR-β, 47-5961; anti-IFN-γ, 25-7311; anti-Foxp3, 17-5773; anti-Vα2, 48-5812). Stained cells were analysed using a LSR-II flow cytometer (Becton Dickinson) and population frequencies were determined using the FlowJo software (Tree Star).

Chromatin immunoprecipitation (ChIP)

20-60 million Th1 polarised cells from WT and dnRara mice were fixed, washed and snap-frozen according to the Cell Fixation protocol from Active Motif (http://www.activemotif.com/documents/1848.pdf). Chromatin was isolated by the addition of lysis buffer, followed by disruption with a Dounce homogenizer. Lysates were sonicated and the DNA sheared to an average length of 300-500 bp. Genomic DNA (Input) was prepared by treating aliquots of chromatin with RNase, proteinase K and heat for de-crosslinking, followed by ethanol precipitation. Pellets were resuspended and the resulting DNA was quantified on a NanoDrop spectrophotometer. Extrapolation to the original chromatin volume allowed quantitation of the total chromatin yield. An aliquot of chromatin was precleared with protein A agarose beads (Invitrogen). Following immunoprecipitation with specified antibodies, complexes were washed, eluted from the beads with SDS buffer, and subjected to RNase and proteinase K treatment. Crosslinks were reversed by incubation overnight at 65 C, and ChIP DNA was purified by phenol-chloroform extraction

and ethanol precipitation and used for the preparation of Illumina sequencing libraries and for ChIP qPCR analysis.

ChIP-qPCR

Quantitative PCR (qPCR) reactions were carried out in triplicate on specific genomic regions using SYBR Green Supermix (Bio-Rad). See Table S5 for Primer details. The resulting signals were normalized for primer efficiency by carrying out qPCR for each primer pair using Input DNA. By using standards of known quantities of DNA it was possible to calculate the number of genome copies pulled down for each of the sites tested, and thus to calculate the copies pulled down per starting cell number, presented as 'Enrichment'. For RARα ChIP qPCR a gene desert on chromosome 6 (Untr6) was used for a negative control site (Active Motif Catalog No: 71011).

ChIP Sequencing (Illumina)

Illumina sequencing libraries were prepared from the ChIP and Input DNAs using standard procedures and libraries were sequenced on HiSeq 2500

ChipSeq Analysis

For each sample the 50bp SE reads in FastQ format from the sequencer were aligned to the mouse reference genome (mm10) using Novoalign v2.07.11 (http://www.novocraft.com). The resulting alignment file was converted to BAM format using samtools (http://samtools.sourceforge.net/) and the pcr duplicates were removed using picard tools (http://picard.sourceforge.net). Only uniquely mapped reads from each

sample were selected for further analysis. Significantly enriched regions from each sample were identified with MACS v2.0.10_20131216 (Zhang et al. 2008, Feng J et al. 2011) (with q=0.10) using the input sample for background correction. In some instances peaks were identified by visual inspection and confirmed by ChIP qPCR. In case of H3K4me1 and H3K27me3 samples, “--broad” setting was used to merge nearby enriched regions. For visualization purposes, the input signal was subtracted from each ChIP sample and was converted into bigWig format using “bedGraphToBigWig“ utility from UCSC tools (http://genome.ucsc.edu/util.html). The identified significantly enriched regions were annotated to find the associated genes using “FindNeighbouringGenes” utility from USeq package (http://useq.sourceforge.net/). Associated genes represent the closest transcriptional start site from the centre of the peak.

Microarray data

Total RNA was extracted from cells lysed in Trizol LS reagent (Life Technologies). RNA quality was assessed with an Agilent 2100 Bioanalyzer (Agilent Technologies) and quantified with the Nanodrop ND-1000 UV-spectrophotometer (NanoDrop Technologies).

Transcriptome in IFN-γ^+ (eYFP$^+$) CD4$^+$ T-cells

Naïve CD4$^+$ T-cells from dn*Rara*-IFN-γ^{eYFP} or littermate control IFN-γ^{eYFP} reporter mice were cultured under Th1 conditions. On day 7 of culture, following restimulation with PMA and ionomycin, eYFP$^+$ cells were sorted and total RNA was extracted for transcriptional profiling using Affymetrix Mouse Gene 2.0 ST arrays. Pre-processing and statistical analysis of gene expression data were done using Partek Genomics Suite 6.6. CEL files

were imported and expression intensities were summarised, normalised and transformed using Robust Multiarray Average algorithm. Two additional samples from eYFP$^+$ dn*Rara* or wild-type cells sorted without prior restimulation were included in the normalisation. These samples were not included in the analysis of differentially expressed genes. P values <0.05 and fold change in expression ≥ 1.5 or ≤ -1.5 were considered significant.

<u>Transcriptome in Th1 differentiated cells</u>

Sorted naïve CD4$^+$ T-cells from dn*Rara* or WT mice were polarised under Th1 conditions. On day 6 of culture cells were harvested and total RNA was extracted for microarray study or ChIP. RNA isolation, microarray and data processing performed by Miltenyi Biotec. Transcriptome analysis was performed using Agilent Whole Mouse Genome Oligo Microarrays 8X60K in accordance with manufacturer's protocol. Data analysis was performed using R/bioconductor and software packages therein (http://www.R-project.org ; http://www.bioconductor.org) or MS-Office Excel (Microsoft Inc.). Background corrected intensity values were normalized between arrays using quantile normalization. Quality controls include comparison of intensity profiles and a global correlation analysis. Differentially expressed genes were identified by statistical group comparisons on normalized (background corrected and quantile normalized) log2 transformed fluorescence intensities using Student´s t-test (two-tailed, equal variance). Reporters showing a p-value ≤ 0.05 and a median fold-change in expression ≥ 1.5 or ≤ -1.5 were considered as reliable candidates for altered gene expression. In addition, at least two of the replicate samples in the group with higher expression were required to have detection p-values ≤ 0.01.

Supplemental References

Feng, J., Liu, T. and Zhang, Y. (2011) Using MACS to Identify Peaks from ChIP-Seq Data. Current Protocols in Bioinformatics. 34:2.14:2.14.1–2.14.14.

Mucida, D., Park, Y., Kim, G., Turovskaya, O., Scott, I., Kronenberg, M., Cheroutre, H. (2007) Reciprocal TH17 and Regulatory T Cell Differentiation Mediated by Retinoic Acid. Science *317*, 256-260

Yong Zhang, Tao Liu1, Clifford A Meyer, Jérôme Eeckhoute, David S Johnson, Bradley E Bernstein, Chad Nusbaum, Richard M Myers, Myles Brown, Wei Li7 and X Shirley Liu. (2008) Model-based Analysis of ChIP-Seq (MACS). Genome Biology. 9:R 137
