# Supplementary material for: Retinoic Acid Is Essential for Th1 Cell Lineage Stability and Prevents Transition to a Th17 Cell Program
Source: Immunity. 2015 Mar 17;42(3):499–511. doi: 10.1016/j.immuni.2015.02.003 (PMC4372260; doi:10.1016/j.immuni.2015.02.003)
Supplement: Document S2. Article plus Supplemental Information [file mmc2.pdf]

Immunity

Article

Retinoic Acid Is Essential for Th1 Cell Lineage Stability and Prevents Transition to a Th17 Cell Program

Graphical Abstract

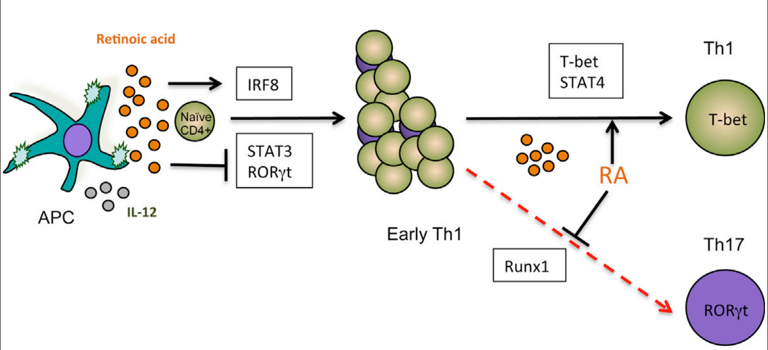

Highlights

- Retinoic acid (RA) stabilizes Th1 fate commitment
- Signaling through RA receptor α (RARα) activates enhancers of Th1-cell-lineage-specifying genes
- RA-RARα represses Th17-cell genes in Th1 cells and constrains Th1-cell plasticity
- RA-RARα prevents development of pathogenic Th17 cells in vivo

Authors

Chrysothemis C. Brown, Daria Esterhazy, ..., Graham M. Lord, Randolph J. Noelle

Correspondence

chrysothemis.brown@kcl.ac.uk (C.C.B.), rjn@dartmouth.edu (R.J.N.)

In Brief

Maintenance of T helper (Th)-cell identity is critical for appropriate immune responses; however, the factors that regulate Th-cell plasticity are unresolved. Brown et al. show that retinoic-acid signaling confers Th1 cell stability and restrains their conversion to Th17 cells.

CrossMark
Brown et al., 2015, Immunity *42*, 499–511
March 17, 2015 ©2015 The Authors
http://dx.doi.org/10.1016/j.immuni.2015.02.003

CellPress

CellPress

Retinoic Acid Is Essential for Th1 Cell Lineage Stability and Prevents Transition to a Th17 Cell Program

Chrysothemis C. Brown,[1,*] Daria Esterhazy,[2] Aurelien Sarde,[1] Mariya London,[2] Venu Pullabhatla,[3] Ines Osma-Garcia,[1] Raya al-Bader,[1] Carla Ortiz,[1] Raul Elgueta,[1] Matthew Arno,[4] Emanuele de Rinaldis,[3,5] Daniel Mucida,[2] Graham M. Lord,[1,3] and Randolph J. Noelle[1,6,*]
[1]Division of Transplantation Immunology and Mucosal Biology, King's College London, London SE1 9RT, UK
[2]Laboratory of Mucosal Immunology, The Rockefeller University, New York, NY 10065, USA
[3]National Institute for Health Research Biomedical Research Centre at Guy's & St Thomas' National Health Service Foundation Trust and King's College London, London SE1 9RT, UK
[4]Genomics Centre, King's College London, London SE1 9NH, UK
[5]Division of Cancer Studies, School of Medicine, King's College London, Guy's Hospital, London SE1 9RT, UK
[6]Department of Microbiology and Immunology, Dartmouth Medical School, Lebanon, NH 03756, USA
*Correspondence: chrysothemis.brown@kcl.ac.uk (C.C.B.), rjn@dartmouth.edu (R.J.N.)
http://dx.doi.org/10.1016/j.immuni.2015.02.003
This is an open access article under the CC BY license (http://creativecommons.org/licenses/by/4.0/).

SUMMARY

CD4$^+$ T cells differentiate into phenotypically distinct T helper cells upon antigenic stimulation. Regulation of plasticity between these CD4$^+$ T-cell lineages is critical for immune homeostasis and prevention of autoimmune disease. However, the factors that regulate lineage stability are largely unknown. Here we investigate a role for retinoic acid (RA) in the regulation of lineage stability using T helper 1 (Th1) cells, traditionally considered the most phenotypically stable Th subset. We found that RA, through its receptor RARα, sustains stable expression of Th1 lineage specifying genes, as well as repressing genes that instruct Th17-cell fate. RA signaling is essential for limiting Th1-cell conversion into Th17 effectors and for preventing pathogenic Th17 responses in vivo. Our study identifies RA-RARα as a key component of the regulatory network governing maintenance and plasticity of Th1-cell fate and defines an additional pathway for the development of Th17 cells.

INTRODUCTION

Functional plasticity within cells of the innate and adaptive immune system increases the breadth of response to pathogens while also limiting responses detrimental to the host. CD4$^+$ T cells diversify into distinct effector subsets upon antigenic stimulation. Cytokines and other microenvironmental factors present during T-cell priming direct differentiation via induction of lineage specifying transcription factors (TFs): T-bet is the "master" regulator for T helper 1 (Th1) cells, RORγt for Th17 cells, and GATA3 directs the Th2 program. In vivo, the presence of cells that express TFs and cytokines from opposing Th lineages indicates flexibility between those subsets. Late-stage developmental plasticity is potentially perilous: interferon-γ (IFN-γ$^+$) Th17 cells have been implicated in several human autoimmune diseases including inflammatory bowel disease (Annunziato et al., 2007), juvenile idiopathic arthritis (Nistala et al., 2010), and multiple sclerosis (Kebir et al., 2009); ex-Foxp3$^+$ Th17 cells play a pathogenic role in rheumatoid arthritis (Komatsu et al., 2014); and interleukin-17 (IL-17$^+$) Th2 cells have been positively linked to the severity of asthma (Irvin et al., 2014). Elucidating the developmental pathways for these hybrid cells and identifying the factors that regulate Th-cell stability are therefore of critical importance.

Initial lineage specification is driven by cytokines, which activate signal transducer and activator and transcription (STAT) proteins: expression of T-bet is driven by IFN-γ-STAT1 and IL-12-STAT4 (Schulz et al., 2009); RORγt by STAT3 downstream of IL-6, IL-21, and IL-23 (Zhou et al., 2007). Less is known about the molecular mechanisms that sustain lineage identity. Epigenetic modifications stabilize gene expression and as such, are thought to play a key role in the maintenance of cell-fate commitment. However, the factors that co-ordinate chromatin changes with evolving TF networks in differentiating Th cells are not fully defined. One candidate is the vitamin A metabolite, retinoic acid (RA). RA is known to play a key role in directing the lineage fate of hematopoietic stem cells (Chanda et al., 2013), dendritic cells (DCs) (Klebanoff et al., 2013), innate lymphoid cells (ILCs) (Spencer et al., 2014), and CD4$^+$ T cells (Reis et al., 2013) through activation of the nuclear RA receptor (RAR). In addition to its classical role as a transcriptional regulator, recent studies in embryonic stem cells have identified RA-RAR as an epigenetic regulator (Kashyap et al., 2013; Urvalek and Gudas, 2014). RA synthesis is dynamically controlled at sites of T-cell priming during inflammation, where RA signaling on T cells has been demonstrated (Aoyama et al., 2013; Pino-Lagos et al., 2011). These studies suggest a potential role for RA in Th-cell plasticity. Indeed, RA is critical for Th1-cell immunity (Hall et al., 2011; Pino-Lagos et al., 2011) and RA has also been implicated in Th17-cell differentiation where its impact appears to be

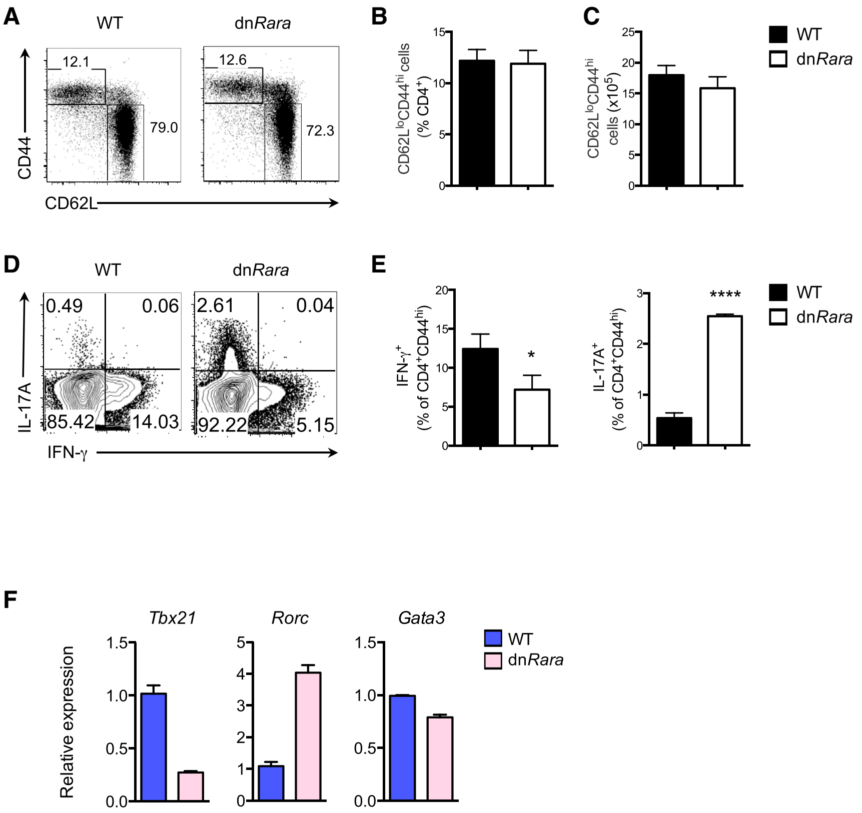

Figure 1. RA Controls the Balance between Th1 and Th17 Effector Cells

(A) Splenic CD4$^+$ T cells from dn*Rara* and WT littermate control mice. Numbers indicate percentage CD62L^{lo}CD44^{hi} cells (top left) or CD62L^{hi} CD44^{lo} T cells (bottom right) gated on CD4$^+$ cells.

(B) Frequency and total number (C) of CD62L^{lo}CD44^{hi} in the CD4$^+$ T-cell population in WT and dn*Rara* mice (n = 3 or 4 per group).

(D) Intracellular IFN-γ and IL-17A expression in splenic CD4$^+$CD44^{hi} T cells after stimulation with phorbol 12-myristate 13-acetate (PMA) and ionoymycin.

(E) Statistical data from cells as in (D).

(F) Quantitative real-time PCR analysis of *Tbx21*, *Rorc*, and *Gata3* in splenic CD4$^+$CD62^{lo}CD44^{hi} cells (as in 1A), sorted by flow cytometry.

Data are from two or three independent experiments with similar results. Mean ± SEM, *p < 0.05; ****p < 0.0001

See also Figure S1.

dose dependent: physiological concentrations of RA enhance Th17-cell differentiation in vitro (Takahashi et al., 2012), yet administration of higher concentrations of RA both in vitro and in vivo negatively regulates Th17-cell responses (Mucida et al., 2007; Takahashi et al., 2012; Xiao et al., 2008). Although RARα has been identified as the critical mediator of RA actions in CD4$^+$ T cells (Hall et al., 2011), to date a comprehensive analysis of the transcriptional targets of RARα in CD4$^+$ T cells has not been reported and the mechanism by which RA regulates these distinct Th-cell fates remains unresolved.

Here we show that RA-RARα is critical for maintenance of the Th1-cell lineage. Loss of RA signaling in Th1 cells resulted in the emergence of hybrid Th1-Th17 and Th17 effector cells. Global analysis of RARα binding and enhancer mapping revealed that RA-RARα directly regulated enhancer activity at Th1-cell-lineage-defining genes while repressing genes that regulate Th17-cell fate. In the absence of RA signaling, infectious and oral antigen induced inflammation resulted in impaired Th1-cell responses with deviation toward a Th17-cell phenotype. These findings identify RA-RARα as a key regulatory node that acts to sustain the Th1-cell response while repressing Th17-cell fate.

RESULTS

RA-RARα Regulates the Balance between Th1 and Th17 Cells

To directly assess the role of RA in Th-cell differentiation in vivo we used mice carrying a sequence encoding a dominant-negative form of the RA receptor RARα (RARα403) targeted to ROSA26 downstream of a *loxP*-flanked "stop" (lsl) cassette. As shown previously (Pino-Lagos et al., 2011), interbreeding with mice expressing Cre recombinase from the *Cd4* promoter generates *Cd4*^{cre}dn*Rara*$^{lsl/lsl}$ progeny (dn*Rara* mice) in which RA signaling is abrogated within the T-cell compartment. In contrast to *Rara*$^{-/-}$ mice, expression of this dnRARα disrupts the RA dependent activity of RARα while retaining the ligand independent effects, allowing the specific analysis of RA-dependent functions.

To investigate the role of RA in the generation of Th-cell subsets under steady-state conditions, we determined the expression of cytokines within CD4$^+$ T cells with an activated CD44^{hi} phenotype. Examination of the peripheral CD4$^+$ T-cell compartment revealed equivalent frequencies and absolute numbers of CD44^{hi}CD62^{lo}CD4$^+$ memory cells in 8-week-old dn*Rara* mice and in Cre$^-$, wild-type (WT), littermate controls (Figures 1A–1C). dn*Rara* effector cells displayed reduced production of IFN-γ compared to their WT counterparts with a >5-fold increase in the frequency of IL-17$^+$ cells (Figures 1D and 1E). Examination of transcripts for the signature lineage-determining TFs showed reduced mRNA expression of *Tbx21* and significantly higher expression of *Rorc* in dn*Rara* effector CD4$^+$ T cells (Figure 1F). Loss of RA signaling had no impact on Th2 effectors with equivalent levels of *Gata3* expression between dn*Rara* and WT mice (Figure 1F) and similar frequencies of IL-4 producing CD4$^+$ T cells (data not shown).

The frequency and numbers of Foxp3$^+$ T cells in the periphery and thymus of dn*Rara* mice were similar to control mice (Figures S1A and S1B), indicating that the increase in Th17 cells was not a consequence of reciprocal regulation by RA of Foxp3$^+$CD4$^+$ T cells and Th17 cells (Mucida et al., 2007). Therefore it is likely that under steady-state conditions RA is critical for differentiation of Th1 cells, while also limiting the differentiation of Th17 cells.

RA Promotes Th1-Cell Differentiation and Inhibits Development of Th17 Cells from Th1 Cell Precursors

We considered two alternative explanations of why dn*Rara* mice exhibit reduced memory effector Th1 cells, in parallel with enhanced Th17 cells. The first possibility was that RA is required for the development of Th1 cells while independently suppressing the primary differentiation of Th17 cells. The alternative possibility was that RA is critical in restraining conversion of Th1 cells to Th17 cells. In order to resolve these two possibilities, naive $CD4^+$ T cells were differentiated in the presence of Th1 or Th17 polarizing cytokines. dn*Rara* expressing $CD4^+$ T cells differentiated under Th1 cell conditions showed a markedly reduced capacity for IFN-γ production (Figure 2A). Diminished cytokine production was not a consequence of impaired proliferative responses as naive $CD4^+$ T cells differentiated under Th1-cell conditions showed robust proliferation, equivalent to WT cells (Figure S2A). In addition, upregulation of the activation markers CD25 and CD44 indicated that dn*Rara* T cells were not impaired in their ability to differentiate into effector cells (Figure S2B). Analysis of TF expression showed that ablating RA signaling resulted in a dramatic reduction in the expression of T-bet in $CD4^+$ T cells differentiated under Th1-cell conditions (Figure 2B). Strikingly, a substantial proportion of dn*Rara* Th1 cells expressed RORγt and co-expression of T-bet and RORγt was observed at the single-cell level. Although we did not observe intracellular IL-17A in cells following brief stimulation with phorbol myristate (PMA) and ionomycin, analysis of supernatants from Th1 polarized cells, reactivated on day 6 of culture on anti-CD3 and anti-CD28 coated plates for 24 hr in non-polarizing media, showed increased expression of IL-17A alongside other Th17-cell-associated cytokines (IL-21 and IL-22) (Figure 2C). Furthermore, mRNA analysis of dn*Rara* Th1 polarized cells revealed dramatic increases in expression of key signature Th17-cell genes (Figure 2D). Notably, these Th1 cells displayed the hallmarks of pathogenic Th17 cells with high amounts of *Il23r* expression but reduced amounts of IL-10 mRNA and protein (Figures 2C and 2D) (Basu et al., 2013).

In order to assess whether enhanced Th17 responses were a general feature of $CD4^+$ T cells in which RA signaling is disrupted, naive $CD4^+$ T cells from dn*Rara* mice were differentiated under Th17 polarizing conditions. In contrast to our observations above, we did not observe an increase in the frequency of IL-17^+ cells in dn*Rara* mice during primary differentiation into Th17 cells (Figure S2C), suggesting that RA restrains Th17-cell differentiation only in the context of a Th1 polarizing cytokine milieu. In support of this, RORγt expression was not observed in dn*Rara* expressing naive $CD4^+$ T cells differentiated under Th0 or Th2 conditions (Figure S2D).

The simultaneous expression of RORγt and T-bet in dn*Rara* Th1 cells suggested that RA-RARα might act to constrain the deviation of Th1 committed cells toward the Th17-cell lineage. To determine whether the $\text{ROR}\gamma\text{t}^+$ cells represented a distinct T-cell population that arose directly from naive $CD4^+$ T cells or from previously committed Th1 cells, we interbred *Ifng*^{eYFP} (Great) reporter mice with the dn*Rara* mice to allow the tracking of IFN-γ^+ cells. Naive $CD4^+$ T cells from dn*Rara-Ifng*^{eYFP} or littermate control mice were activated under Th1 polarizing conditions. On day 7 of culture, eYFP^+ (IFN-γ^+) cells were FACS sorted and underwent genome-wide expression analysis. Key signature Th17-cell genes, including Th17-cell cytokines and receptors for cytokines that promote Th17-cell differentiation (*Il17f*, *Il21*, *Il1r1*, *Il6ra*, and *Il23r*), were highly expressed in dn*Rara* IFN-γ expressing cells relative to WT mice, confirming a hybrid Th1-Th17-cell phenotype (Figure 2E). Of note, these Th1-Th17 cells retained high expression of *Il12rb2* and *Cxcr3* mRNA, equivalent to WT Th1 cells, while also expressing *Il23r* (Figure S2E). Genes associated with the Th2-cell subset such as *Gata3* and *Il4* were also dysregulated in dn*Rara* Th1 cells consistent with a role for T-bet in repression of GATA3 (Zhu et al., 2012). These findings show that, in the absence of RA signaling, committed Th1-cell precursors can give rise to cells with a Th17-cell expression signature providing a new perspective on the origins of Th1-Th17 cells. Collectively, these data demonstrate that RA is not only required for Th1-cell differentiation but is also critical in suppressing Th17-cell development in Th1 polarized cells.

RA-RARα Is Required for Late-Phase, STAT4-Dependent T-bet Expression in Th1 Cells

Early expression of T-bet following TCR activation is dependent on IFN-γ, whereas late expression of T-bet (post-termination of TCR signaling) has been shown to be dependent on IL-12 (Schulz et al., 2009). To distinguish a requirement for RA signaling in Th1-cell commitment from maintenance of Th1-cell fate, we examined the kinetics of T-bet expression in naive $CD4^+$ T cells cultured under Th1 polarizing conditions. Induction of T-bet was observed with comparable amounts of T-bet expression between WT and dn*Rara* T cells at day 3 of culture, indicating that RA-RARα signaling is not required for early Th1 lineage commitment (Figure 3A). However, T-bet expression was not sustained in dn*Rara* Th1 cells, with substantially diminished expression of T-bet by day 5 of culture. Given that IFN-γ promotes T-bet expression, the expression of T-bet was examined in the presence of recombinant IFN-γ, in order to avoid potential indirect effects caused by reduced IFN-γ production in dn*Rara* Th1 cells. Exogenous IFN-γ enhanced early T-bet expression in both dn*Rara* and WT Th1 cells but did not rescue the late (>72 hr) impairment in T-bet expression (Figure 3A). IFN-γ signaling, as measured by STAT1 phosphorylation, was not impaired at either time point (data not shown).

The late IL-12-dependent peak of T-bet expression observed in the presence of blocking IFN-γ antibodies was abrogated in dn*Rara* Th1-cell polarized cells (Figure 3A) suggesting impaired STAT4 activity. At day 3 of culture, comparable amounts of phosphorylated STAT4 (pSTAT4) were observed between dn*Rara* and WT mice. By contrast, at day 6 of culture, IL-12 induced pSTAT4 was markedly impaired in dn*Rara* T cells (Figure 3B) despite comparable expression of IL-12Rβ2 mRNA and protein expression and increased expression of *Il12rb1* mRNA (Figure 3C and 3D). Analysis of *Stat4* expression, demonstrated impaired induction of *Stat4* in the absence of RA signaling (Figure 3E) with reduced amounts of total STAT4 protein (Figure 3F). These findings suggest that the observed reduction in pSTAT4 in dn*Rara* Th1 cells is a consequence of diminished STAT4 expression. Consistent with deviation toward the Th17-cell lineage, we observed enhanced pSTAT3 activity in Th1-cell polarized dn*Rara* cells with an increased ratio of pSTAT3/pSTAT4 (Figures S3A and S3B).

To evaluate whether the impairment in T-bet and STAT4 expression correlated with changes in IFN-γ, the time course

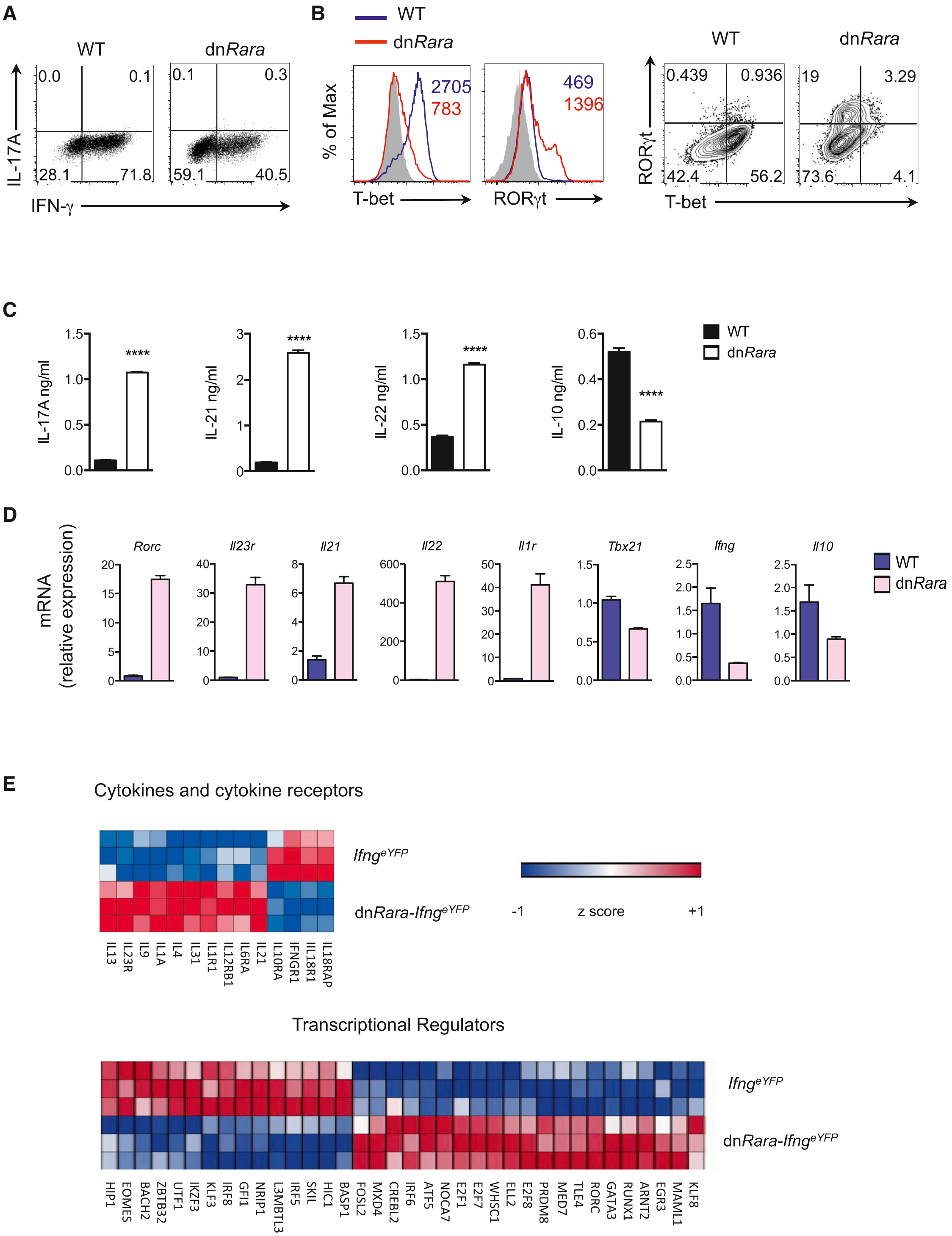

Figure 2. RA Signaling Required for Th1-Cell Differentiation and Repression of Th17-cell Fate in Th1-Cell Precursors

Sorted naive CD4+ T cells from dn*Rara* or WT mice were cultured under Th1 conditions for 6 days.

(A) Intracellular expression of IFN-γ and IL-17A following stimulation with PMA and ionomycin.

(B) T-bet and RORγt expression. Grey histograms indicate staining for *Tbx21*$^{-/-}$ (left panel) or isotype control antibody (right panel). Numbers show MFI. Numbers in quadrants represent percent cells in each.

C) Amount of IL-17A, IL-21, IL-22, and IL-10 in supernatants following restimulation of cells as in (A) with α-CD3 and α-CD28 for 24 hr as measured by multiplex bead array. Triplicate culture wells.

(D) Quantitative real time PCR analysis of Th1 and Th17-cell signature cytokine and TF genes following stimulation with PMA and ionomycin.

(legend continued on next page)

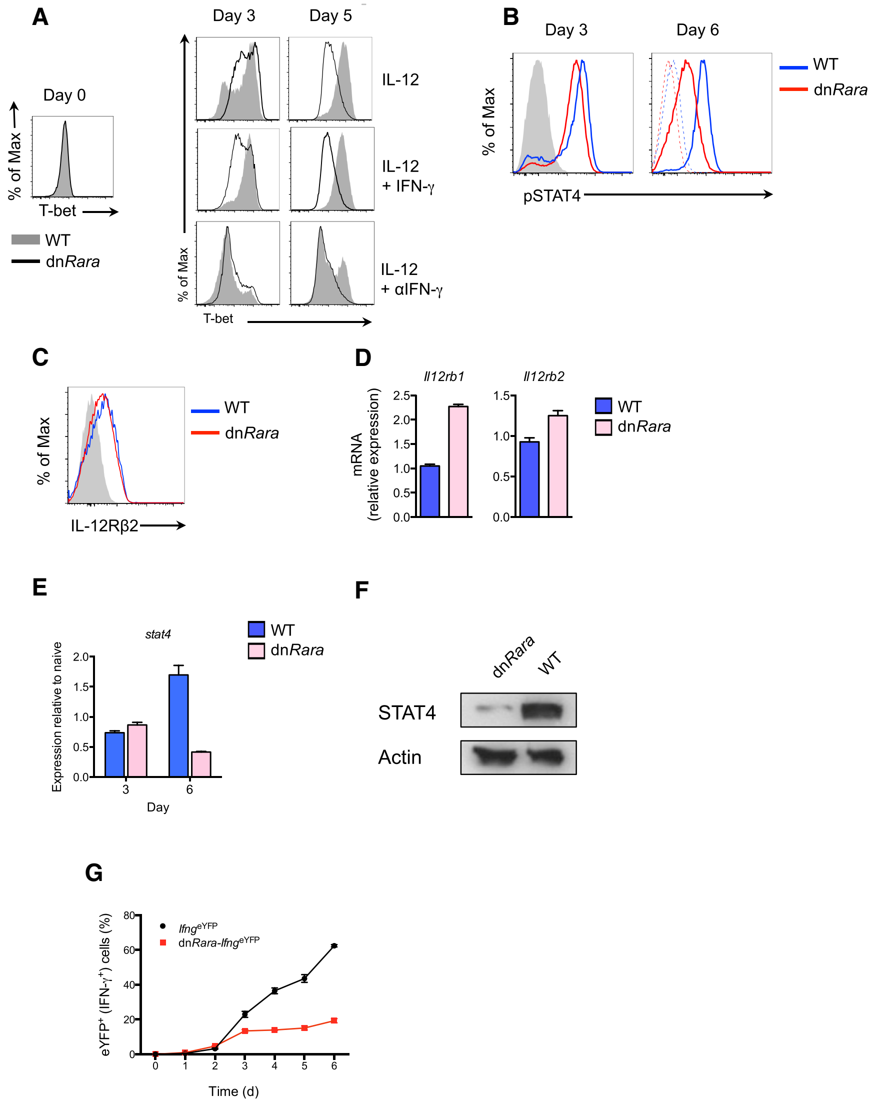

Figure 3. RA Required for Late Phase T-bet Expression

(A) Naive CD4$^+$ T cells from dn*Rara* and WT mice were differentiated under Th1 conditions with combinations of IFN-γ or IFN-γ antibody. T-bet expression analyzed at the indicated time points. Histograms gated on CD4$^+$ T cells.

(B) Flow cytometric analysis of STAT4 phosphorylation in naive CD4$^+$ T cells from dn*Rara* and WT mice differentiated under Th1 conditions. Cells analyzed directly from culture after 3 days (left panel) or on day 6 following treatment with (solid lines) or without (dashed lines) 25 ng/ml IL-12 for 30 min (right panel). Shaded histogram displays pSTAT4 staining in cells cultured under Th0 conditions.

(C) Cell-surface expression of IL-12Rβ2 on day 6 of culture.

(D) Quantitative real-time PCR analysis of *Il12rb1 and Il12rb2* on day 6.

(E) Quantitative real-time PCR analysis of *Stat4* in Th1 polarized cells at indicated time points. Expression relative to naive CD4$^+$ T cells.

(F) Western blot analysis of total STAT4 protein on day 6 of Th1 culture.

(G) Naive CD4$^+$ T cells from dn*Rara-Ifng*^{eYFP} and control mice were activated under Th1 conditions. Frequency of IFN-γ$^+$ (eYFP$^+$) cells at indicated time points, gated on viable CD4$^+$.

Data representative of two to three independent experiments. Mean ± SEM.

See also Figure S3.

of IFN-γ expression following initiation of Th1-cell polarization was analyzed in naive dn*Rara-Ifng*^{eYFP} expressing CD4$^+$ T cells. The kinetics of IFN-γ induction, as measured by frequency of eYFP$^+$ cells, closely mirrored WT cells during the first 72 hr of culture but expression was not sustained in the absence of RA signaling (Figure 3G). Collectively, these data show that RA plays a temporal role in Th1 differentiation, maintaining Th1-cell commitment through regulation of T-bet and STAT4.

RA-RARα Regulates Th1-Cell Plasticity

Alterations in the stable expression of lineage-determining TFs are thought to underlie Th-cell stability or plasticity. The emergence of Th1-Th17 cells together with the loss of T-bet expression, suggested a role for RA in the regulation of Th1-cell plasticity. However, diminished T-bet and STAT4 activity from day 3 of primary Th1-cell differentiation prevented assessment of lineage stability in fully differentiated Th1 cells. To determine whether RA-RARα was required for long-term Th1-cell fate, we differentiated naive CD4$^+$ T cells from dn*Rara*$^{lsl/lsl}$ mice under Th1-cell conditions, treated them with TAT-Cre (Wadia et al., 2004) on days 5 and 7, and restimulated them under Th1-cell conditions for a further 5 days. The temporal loss of RA signaling in Th1 cells resulted in decreased T-bet expression with a reciprocal increase in RORγt expression (Figure 4A). ~50% of cells expressed RORγt, which suggests that ongoing RA-RARα activity is critical for sustaining T-bet and suppressing Th17-cell fate. Alterations in the lineage determining TFs did not impact on the cytokine phenotype (Figure S4A). This might in part reflect T-bet independent regulation of the *Ifng* locus at late stages in Th1-cell development.

To further examine the role of RA in Th1-cell stability, naive CD4$^+$ T cells from *Ifng*^{eYFP} mice were differentiated under

(E) Naive CD4$^+$ T cells from dn*Rara-Ifng*^{eYFP} and *Ifng*^{eYFP} mice were cultured under Th1 conditions. IFN-γ (eYFP$^+$) cells were sorted on day 7 following stimulation with PMA and ionomycin. Heatmaps displaying the fold changes of genes that were differentially expressed (fold change > 1.5, $p < 0.05$) for selected cytokines or cytokine receptors (upper panel) and TFs (lower panel). Samples from three independent experiments.

Representative data of at least three (A and B) or two (C and D) independent experiments. Mean ± SEM.

See also Figure S2.

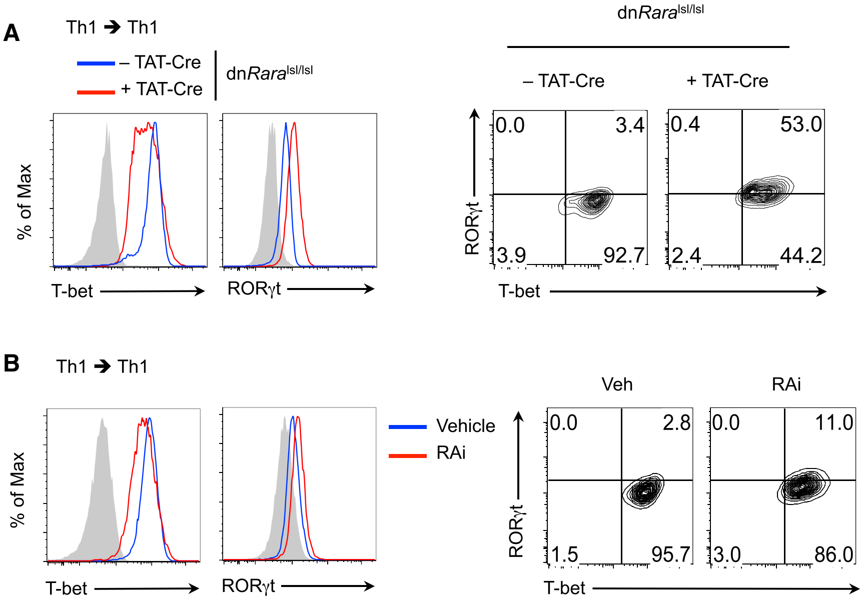

Figure 4. Loss of RA Signaling in Fully Committed Th1 Cells Leads to Th1 Plasticity and Divergence Toward the Th17 Lineage

(A) Naive CD4$^+$ T cells from dn*Rara*$^{lsl/lsl}$ mice were differentiated under Th1 conditions. Th1 cells were transduced with TAT-Cre on days 5 and 7 and repolarized under Th1 conditions for a further 5 days. Intracellular expression of T-bet and RORγt.

(B) Naive CD4$^+$ T cells from *Ifng*^{eYFP} mice were differentiated under Th1 conditions. IFN-γ (eYFP$^+$) cells were sorted on day 7 and restimulated under Th1 conditions for 5 days in the presence of Veh or RAi. Intracellular expression of T-bet and RORγt.

Data representative of two independent experiments.

See also Figure S4.

Th1-cell polarizing conditions. eYFP$^+$ (IFN-γ$^+$) cells were FACS-sorted on day 7 of culture and restimulated under Th1-cell conditions in the presence of the RAR inhibitor LE540 (RAi) or vehicle control (Veh). Inhibition of RA signaling in fully committed Th1 cells propagated for a further 5 days under Th1 conditions resulted in downregulation of T-bet and the emergence of cells co-expressing RORγt (Figure 4B). Diminished T-bet expression was associated with modest reductions in IFN-γ expression (Figure S4B). Taken together, these data establish that loss of RA signaling in fully committed Th1 cells leads to transdifferentiation to progeny with features of the Th17 lineage and support a model where RA constrains late-stage plasticity of Th1 cells.

RA-RARα Regulates Enhancer Activity at Lineage Determining Th1-Cell Genes

To better understand the molecular mechanism by which RARα regulates Th-cell fate, we performed genome-wide analysis of RARα binding in WT Th1 cells by ChIP-Seq, combined with transcriptional profiling of dn*Rara*-expressing Th1 cells in order to identify functional targets of RARα. Selected loci were validated by ChIP-qPCR. RARα binding was identified at 1,766 sites in 1,567 genes. RARα binding was detected at 10.3% (76 of 740 genes) of genes downregulated in the absence of RA signaling (Table S2) (hereafter referred to as positively regulated) and 4.8% (56 of 1,169) of the upregulated genes (Table S3). In keeping with its classical role as a positive regulator of transcriptional activation there was significant enrichment of RARα binding at genes positively regulated by RA (Fisher's exact test, $p < 0.0001$). However, the presence of RARα at a subset of the negatively regulated genes indicates that RA-RARα also plays a role in transcriptional repression within Th1 cells.

RA-RARα-dependent loci included Th1-cell lineage-defining genes (*Tbx21* and *Stat4-Stat1*). In addition to targeting the *Tbx21* promoter (Figures 5A and 5B), modest RARα binding was observed at the conserved T-bet enhancer element, 12kb upstream of the transcriptional start site (TSS) (Yang et al., 2007). This was confirmed by ChIP-qPCR (Figure 5B). Intergenic RARα was also detected at the *Stat4-Stat1* locus and an *Ifng* enhancer element (Figures S5A and S5B).

RA binding to nuclear RARα results in recruitment of co-activator complexes containing the histone acetyl-transferases p300 and CBP (Kamei et al., 1996). p300 is highly enriched at enhancer regions where it acetylates H3K27, a marker of active enhancers (Rada-Iglesias et al., 2011), suggesting a possible role for RA-RARα in regulating enhancer activity. To test this, we mapped genome-wide binding of p300, H3K4me1, H3K4me3, and H3K27ac histone modifications in dn*Rara* and WT Th1-cells, validating selected regions by ChIP q-PCR. Active enhancers were operationally defined as regions with increased intensity of H3K4me1, p300, and H3K27ac with low or absent H3K4me3 (Rada-Iglesias et al., 2011).

RARα binding at the *Tbx21*, *Stat4*, and *Ifng* loci co-localized with p300 binding at enhancer regions (Figures 5A and S5A). dnRARα lacks the activation function 2 (AF2) domain which is required for RA-dependent recruitment of coactivators. Consistent with this, dn*Rara* expressing T cells exhibited a significant reduction in p300 occupancy and H3K27ac deposition at the *Tbx21* enhancer, supporting the direct regulation of enhancer activity by RA-RARα (Figures 5A and 5C). p300 binding at the *Ifng* and putative *Stat4* intergenic enhancers was also dependent on RA-RARα (Figures S5A and S5C). Loss of p300 binding at the *Stat4-Stat1* intergenic enhancer in *dnRara* Th1 cells correlated with reduced *Stat4* transcripts, whereas *Stat1* expression was actually increased, suggesting that this enhancer element regulated *Stat4* transcription. A recent study identified a role for STAT4 in the regulation of Th1 enhancers (Vahedi et al., 2012). Given that STAT4 expression was reduced in dn*Rara* Th1 cells, it was possible that the loss of p300 was in part due to reduced expression of STAT4. To address this issue, we assessed the binding of STAT4 in WT Th1 cells and compared p300 occupancy in WT and *Stat4*$^{-/-}$ Th1 cells using publicly available ChIP-seq data (Table S1) (Vahedi et al., 2012; Wei et al., 2010). Although STAT4 binding was observed at the *Tbx21* enhancer, loss of STAT4 was not associated with obvious differences in p300 binding (Figure S5D), arguing for a direct contribution of RARα to p300 recruitment and enhancer activity. Collectively, these data show that RA regulates expression of key Th1-cell lineage genes through remodeling of enhancer regions.

RA-RARα Represses Th17-Cell Fate in Th1 Cells through Direct Regulation of Th17-Cell Genes

The earlier finding that Th1 cells acquired features of Th17 cells in the absence of RA signaling led us to evaluate direct regulation of Th17-cell-instructing genes by RA-RARα. We first investigated effects of RA on the Th17-cell pioneer factors BATF and IRF4. As previously reported (Basu et al., 2013), these genes were expressed in WT Th1 cells. Strikingly, kinetic analysis of *Batf* and *Irf4* expression in naive cells stimulated under Th1-cell conditions revealed dramatic upregulation of IRF4 (40- to 60-fold) during the initial phase of Th1-cell polarization with comparable expression between dn*Rara* and WT cells (Figure 5D). Loss of RA signaling resulted in derepression of BATF-IRF4 target genes, *Rorc*, *Il23r*, *Il22*, *Il21*, and *Il12rb1* (Figure 5E). This suggested that "balancing" factors must be induced in an RA-dependent manner to restrict the actions of BATF-IRF4 complexes at Th17-cell genes. IRF8, an alternative binding partner for BATF, previously shown to suppress Th17 differentiation (Ouyang et al., 2011), was one of the RARα target genes most suppressed in dn*Rara* Th1 cells. In WT Th1 cells, induction of *Irf*8 expression paralleled *Irf4* expression. However, in dnRara cells *Irf8* expression was not sustained past 24 hr (Figure 5D). RARα bound at a putative upstream enhancer (Figures 5F and 5G) and in the absence of RA signaling, reduced p300 and H3K27ac were observed at this locus (Figure 5H and 5I). Together, these data show that RA directly regulates expression of IRF8 in Th1 differentiating cells and suggests a potential mechanism by which BATF-IRF4 activity is constrained within early Th1 cells.

Transcriptional activation of BATF-IRF4 target genes is dependent on STAT3 and RORγt (Ciofani et al., 2012). Various genes for cytokines and cytokine receptors associated with STAT3 activation (*Il21*, *Il1r1*, *Il6ra*, and *Il23r*) were derepressed in dn*Rara* Th1 cells (Figure 5E). RARα targeted the promoter and an upstream enhancer in the *Il6ra* locus (Figure 5G) with increased H3k27ac observed at the enhancer element in dn*Rara* Th1 cells (Figure 5J). Consistent with this, dn*Rara* Th1 cells failed to downregulate mRNA and cell-surface IL6-Rα expression during Th1 polarization (Figures S5E and S5F). These findings suggest that RA regulates Th1-cell plasticity in part by inhibiting responsiveness to IL-6.

RORγt was not a direct target of RARα. However, disruption of RA signaling resulted in increased expression of *Runx1*, a TF associated with transactivation of *Rorc* (Figure S5E) (Zhang et al., 2008). ChIP analysis confirmed direct regulation of short and long *Runx1* isoform promoters by RA-RARα (Figure 5G). In Th1 cells, the *Rorc* locus is epigenetically silenced by T-bet (Mukasa et al., 2010). However, in dn*Rara* cells, the repressive H3K27me3 mark was reduced at RORγt isoform-specific exon (Figure 5K), consistent with loss of T-bet. These findings suggest that increased RORγt expression in the absence of RARα signaling is in part due to increased accessibility of the *Rorc* locus, with unrestrained activation by Runx1. Collectively these data indicate that RA-RARα antagonises the activity of the core Th17-cell instructing TFs (IRF4, BATF, STAT3, and RORγt), both directly and indirectly, to suppress the Th17-cell gene program. Notably, Th2-cell-associated genes were not identified as targets of RARα (Tables S2 and S3) suggesting that direct repression of alternative cell fates by RA-RARα is specific to the Th17-cell program.

Th1-like Th17 Cells Emerge during Infection with *L. monocytogenes* in the Absence of RA Signaling

To assess the significance of these findings for immune responses in vivo, we intravenously infected WT and dn*Rara* mice with an attenuated strain of *L. monocytogenes* (ΔActA), Lm-2W, which allows tracking of $CD4^+$ T cells specific for listeriolysin O peptide $LLO_{190–201}$ (LLOp). At the peak of the response, $CD4^+$ T cells were isolated from the spleen and LLOp antigen-specific T cells were assayed for expression of cytokines and the TFs, T-bet, and RORγt. dn*Rara* mice mounted an effector-T-cell response of similar magnitude to WT mice with comparable frequencies and total numbers of $CD44^{hi}$LLOp:I-A^b-specific $CD4^+$ T cells (Figures 6A and 6B). In WT mice, Lm-2W induced a Th1-cell restricted response, as evidenced by high T-bet expression within the LLOp-specific T-cell fraction (Figure 6C). LLOp:I-A^{b+} CD4+ T cells from dn*Rara* mice expressed lower amounts of T-bet and a substantial proportion expressed RORγt, with co-expression of these TFs observed in a subset of cells (Figure 6C). At day 7 post-infection, a significant proportion of $CD4^+$ T cells isolated from the spleen of dn*Rara* mice were IL-17^+ or dual IL-$17A^+$IFN-γ^+ with a trend toward reduced frequency of IFN-γ^+ cells (Figure 6D). Measurement of cytokine protein concentrations from splenocytes restimulated with LLOp confirmed reduced amounts of IFN-γ and concomitant increase in IL-17A (Figure S6A). We did not detect IL-4 production by intracellular staining or protein secretion (Figure S6A and S6B). Consistent with our in vitro data showing downregulation of IL6-Rα on WT Th1 cells, cell-surface IL-6Rα was not detectable on WT LLOp:I-A^{b+} $CD4^+$ T cells. However, dn*Rara* LLOp:I-A^{b+} $CD4^+$ T cells retained expression of IL-6Rα (Figure S6C), supporting a potential role for IL-6 signaling in the regulation of Th1-cell plasticity. These findings establish that RA-RARα signaling in T cells constrains the emergence of Th17 cells in a Th1-cell-instructing microenvironment in vivo.

RA Regulates the Th1-Th17-Cell Axis in the Gut and Prevents the Development of Intestinal Inflammation

RA is constitutively synthesized by a subset of DCs in the gut. To address the physiological importance of RA signaling in the regulation of pathogenic intestinal $CD4^+$ T cells, we interbred dn*Rara* mice with OTII mice that transgenically express an ovalbumin (OVA)-specific TCR and transferred naive $CD4^+$ T cells from OTII(dn*Rara*) or WT OTII mice into *Rag1*$^{-/-}$ hosts. Recipients were maintained on an OVA-containing diet for 7 days to induce differentiation within the transferred cells and migration to the intestinal tissue. Consistent with the infection experiments, feeding OTII(dn*Rara*)-recipient mice OVA resulted in a shift in the Th1-Th17-cell balance with a deficiency in IFN-γ-producing cells and increased frequency of IL-17^+ and dual IFN-γ^+IL-17^+ cells in the mesenteric lymph node (MLN), lamina propria lymphocytes (LPL), and spleen (Sp), 7 days after transfer (Figures 7B and 7C). To address the functional significance of the dysregulated cytokine response in dn*Rara* T cells, we orally challenged mice with OVA and evaluated them for development of intestinal inflammation and diarrhea (Figure 7A). Recipients of OTII(dn*Rara*) cells developed accelerated wasting disease relative to mice that received WT OTII cells (Figure 7D). Whereas all of the recipients of OTII(dn*Rara*) cells developed severe diarrhea

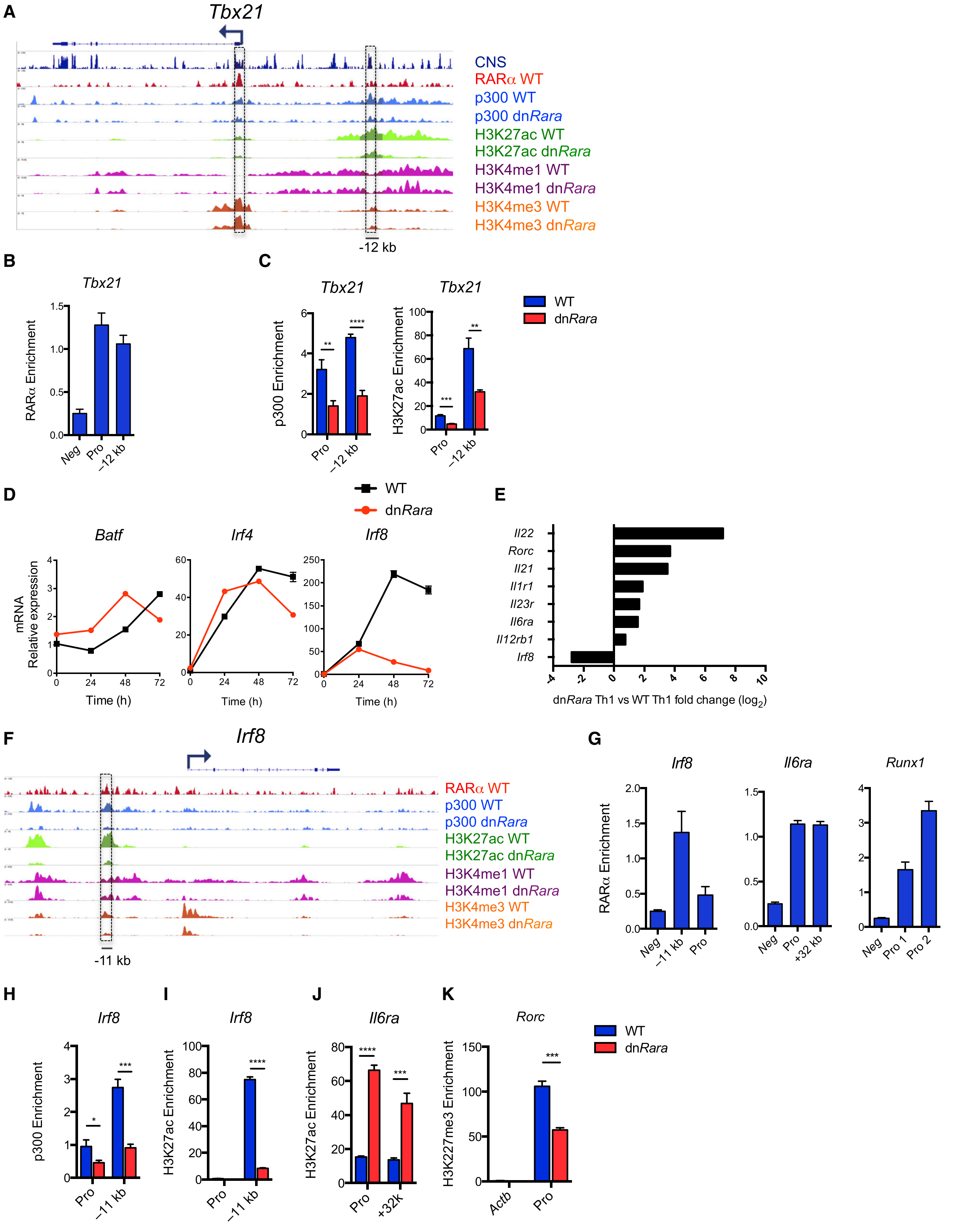

(legend on next page)

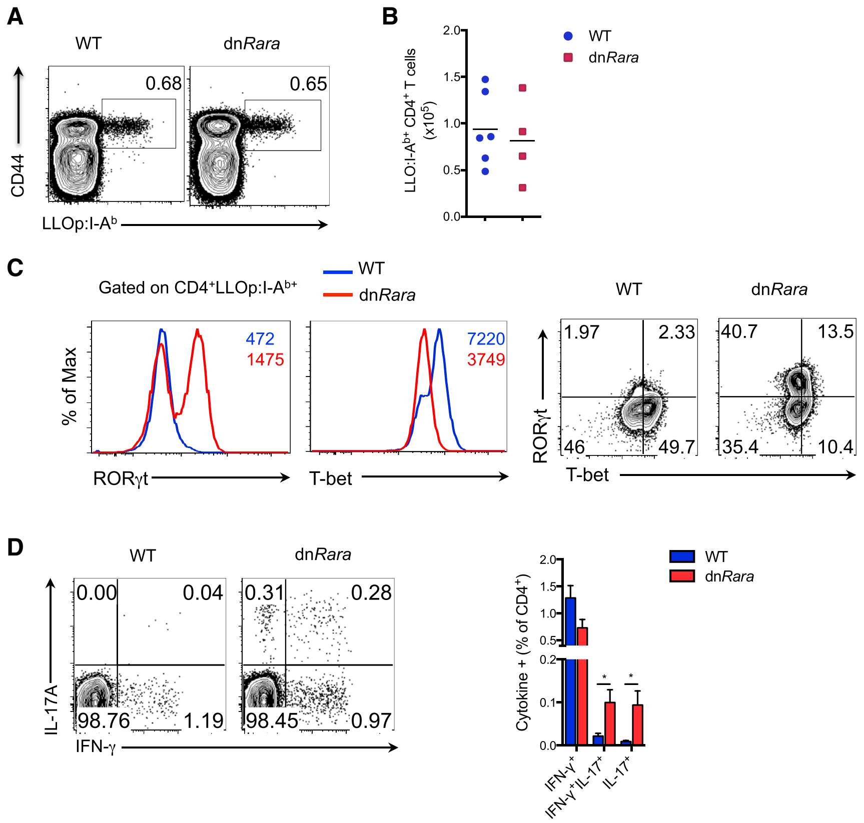

Figure 6. RA Signaling Required to Prevent the Generation of Th17 Cells during Infection with *L. monocytogenes*

(A) Frequency of LLOp:I-A^b CD4$^+$ T cells isolated from spleen of dn*Rara* and WT mice 7 days after infection with an attenuated strain of *L. monocytogenes* (Lm-2W). Gated on CD4$^+$ T cells.

(B) Absolute numbers of LLOp:I-A^b CD4$^+$ T cells as in (A).

(C) Intracellular T-bet and RORγt expression gated on LLOp:I-A^b CD4$^+$ T cells.

(D) Intracellular staining for IFN-γ and IL-17A following stimulation of splenocytes with LLOp for 6 hr, 7 days after infection with Lm-2W. Gated on CD4$^+$ T cells. Right panel shows statistical data pooled from three independent experiments (3–6 mice per group).

Representative data of at least three (A and B), or two independent experiments (C). Mean ± SEM.

See also Figure S6.

by day 12 (Figure 7E), recipients of WT cells remained diarrhea free. Cytokine production was also assessed after the first gavage and confirmed an increased frequency of IL-17$^+$ cells with concomitant reduction in IFN-γ$^+$ cells. Notably, enhanced IL-17 responses were not a consequence of impaired Foxp3$^+$ conversion (Figure 7F). Homing of transferred cells to the gut was not affected in this model with similar frequencies of CD4$^+$ T cells detected in the gut tissues (Figure S7A). We conclude that loss of RA signaling leads to deviation from Th1 to Th17 phenotype both in the periphery and the gut where these Th17 cells are associated with significant intestinal inflammation.

DISCUSSION

Dysregulated Th-cell responses underlie the pathogenesis of autoimmune and allergic disease. In contrast to T regulatory (Treg) cells and Th17 cells, the Th1-cell lineage is thought to be relatively stable. However, the factors that control maintenance of the Th1-cell lineage were not previously known. This study identifies RA-RARα as a central regulatory node in the transcriptional network governing Th1-cell stability. We found that RA-RARα directly sustained the expression of lineage determining Th1-cell-associated genes during naive T-cell differentiation while also repressing signature Th17-cell-associated genes. Ablation of RA signaling in Th1-committed cells resulted in enhanced Th1-cell plasticity with deviation towards a Th17-cell phenotype. Using ChIP-seq to identify regulatory elements, we found that RARα bound at enhancers and recruitment of p300 to these regions was dependent on RA signaling. In vivo, both Th17 and Th1-Th17 cells emerged during infection with *L. monocytogenes* and in a model

Figure 5. RA-RARα Regulates Enhancer Activity at Th1 Lineage Associated Loci and Represses Th17 Genes

Naive CD4$^+$ T cells from WT and dn*Rara* mice were cultured for 6 days under Th1 conditions prior to chromatin precipitation and transcriptional profiling.

(A) ChIP-seq binding tracks at *Tbx21* locus for RARα in WT Th1 cells and p300 binding, H3K27ac, H3K4me1, and H3K4me3 modifications in WT and dn*Rara* Th1 cells.

(B) Validation of the RARα-binding regions in WT Th1 cells by ChIP-qPCR. Untr6 region serves as a negative control. Binding events per 1,000 cells displayed as "Enrichment."

(C) The effects of dn*Rara* expression on p300 and H3k27ac abundance at the Tbx21 locus were validated by ChIP-qPCR.

(D) Quantitative real-time PCR analysis of *Batf*, *Irf4*, and *Irf8* mRNA in naive CD4$^+$ T cells from dn*Rara* or WT cells differentiated under Th1-cell conditions for 0, 24, 48, 72 hr. Mean ± SEM, replicate wells.

(E) Log2 values of fold changes in gene expression as measured by microarray analyses. Average fold change depicted.

(F) ChIP-seq binding tracks at *Irf8* locus for cells as in (A).

(G) Validation of RARα ChIP-seq regions by ChIP-qPCR.

(H–J) ChIP analysis of p300 and H3K27ac at selected loci.

(K) ChIP analysis of H3K27me3 at the RORc locus. *Actb* locus serves as a negative control.

Data from three independent experiments (E) or representative of two independent experiments (B–D, G–K); Mean ± SD unless noted otherwise. Abbreviation: pro, promoter.

See also Figure S5.

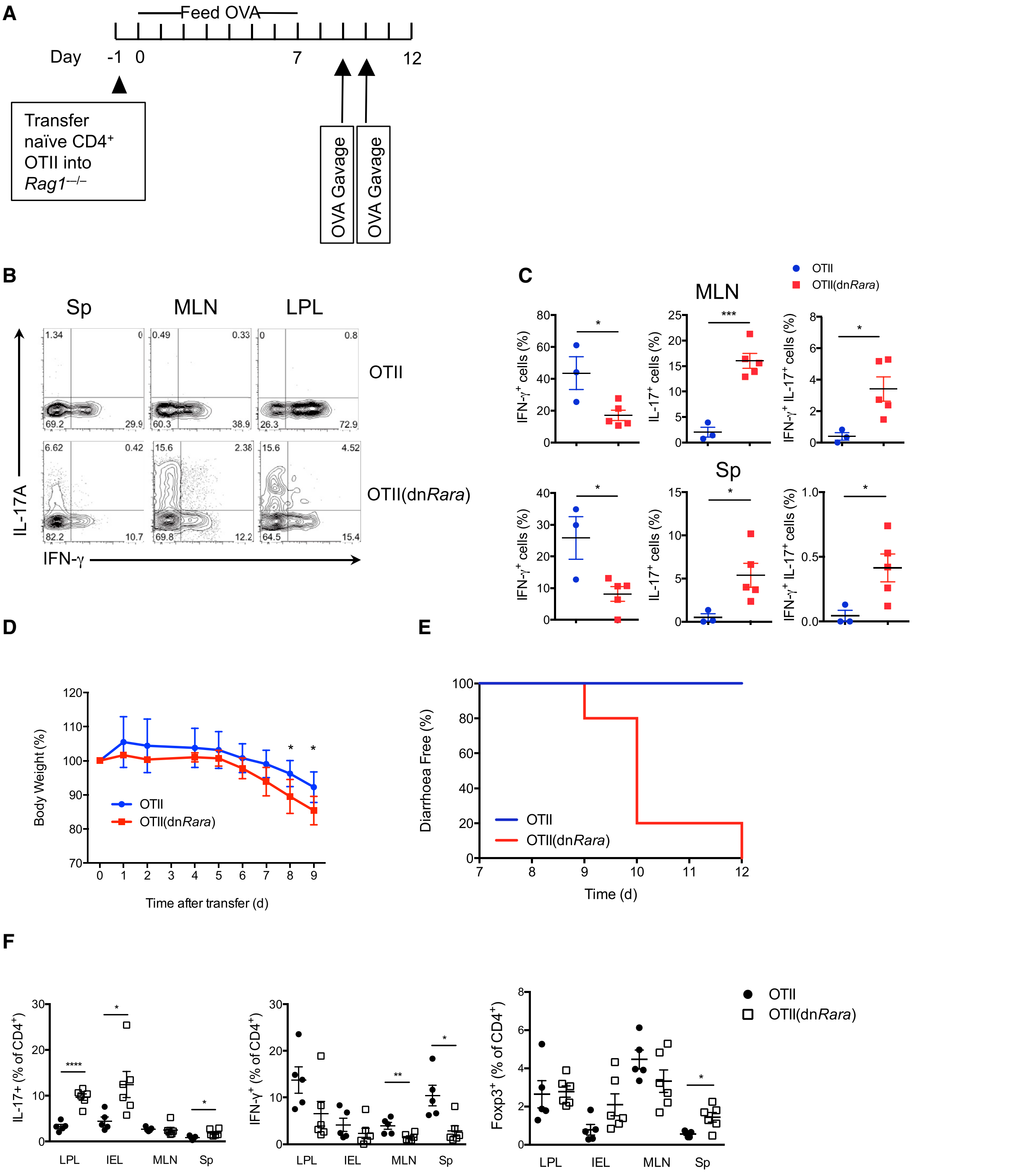

Figure 7. Loss of RA Signaling Causes Dysregulated Th1 and Th17 Response and Increased Pathogenicity in a Model of Gut Inflammation

(A) Schematic illustration of the adoptive transfer experiment.

(B) Intracellular expression of IL-17A and IFN-γ among $CD4^+$ cells from the spleen (Sp), mesenteric lymph nodes (MLN), and lymphocytes from the lamina propria (LPL) of mice as in (A) 7 days after transfer.

(C) Statistical data for frequency of IFN-γ^+, IL-17^+, and IFN-γ^+IL-17^+ cells as in (B) in MLN and Sp.

(D) Percentile change of original body weight in *Rag1*$^{-/-}$ recipients treated as in (A) (n = 5–7 per group). Mean ± SD.

(E) Frequency of diarrhea-free mice among *Rag1*$^{-/-}$ recipients as in (A) (OTII recipients n = 3, OT-II(dn*Rara*) recipients n = 5).

(F) Frequencies of IL-17, IFN-γ, and Foxp3 in $CD4^+$ cells isolated from Sp, MLN, LPL, and IELs of mice as in (A), 9 days after transfer (n = 5 or 6 per group). Data from one experiment (B and C), pooled from two independent experiments (D and F), or representative of two independent experiments (E). Mean ± SEM unless otherwise noted.

of oral tolerance. In the latter, their presence was associated with significant pathology.

Enhancers play a key role in directing cell fate through the regulation of lineage specifying genes. Enhancer profiling in WT and dn*Rara* T cells revealed RA-dependent activation of enhancers at genes critical for Th1 identity (*Tbx21*, *Stat4*, *Ifng*, and *Irf8*). RA-dependent changes in p300 and H3K27ac were reflected at the transcriptional level suggesting that, in addition to its classical role as a transcriptional regulator, RA regulates gene expression in an enhancer-dependent manner. Although the ability of RA-RARα to target p300-CBP complexes to nucleosomes is well established, regulation of enhancers by RA has not been widely studied. We propose that unliganded RARα at enhancer elements acts as a gatekeeper, enabling initiation of enhancer activation once T cells sense RA in the microenvironment. A similar role has been demonstrated for STAT proteins (Vahedi et al., 2012), suggesting that environmental cues act as checkpoints for initiation of enhancer activation and T-cell fate. Although H3K4me1 modifications are present at early time points during T-cell differentiation, conversion to "active" status requires acquisition of H3K27ac, which is often not evident until later stages of differentiation (Hawkins et al., 2013). Consistent with a temporal role for enhancers in maintenance of gene expression, RA signaling was not required for initiation of transcription of target genes but rather acted to maintain their expression. These data highlight the importance of enhancers in maintenance of cell identity and plasticity. It is possible that RA-RARα regulation of enhancers represent the major mechanism by which RA regulates cell fate. A recent study identified enrichment of RARα at enhancers in embryonic stem cells (Chen et al., 2012). Given that the RA-RARα axis is a highly conserved signaling pathway, which plays a critical role in regulating cell-fate specification during embryogenesis and cell differentiation, it will be important to evaluate a broader role for RA-RARα in regulation of enhancer functionality, both in alternative Th-cell subsets and outside of the immune system.

In addition to sustaining expression of Th1-cell-associated genes, we found that RA actively silences genes implicated in Th17-cell differentiation. Among genes known to regulate the Th17-cell program, *Runx1* and *Il6ra* were directly repressed by RA-RARα. In addition, BATF-IRF4 target genes were derepressed in the absence of RA signaling. In Th17 cells, BATF-IRF4 complexes act co-operatively as pioneer factors at key Th17 genes (Ciofani et al., 2012), modulating chromatin accessibility to facilitate binding of STAT3 and RORγt. On the basis of their expression in alternative Th-cell subsets, it has been suggested that BATF-IRF4 complexes play a universal role in establishing binding of lineage-specific TFs (Ciofani et al., 2012). However, BATF deficiency does not impact on Th1-cell differentiation (Schraml et al., 2009). An alternative model is that upregulation of BATF and IRF4 confers plasticity in early Th1 cells, poising chromatin specifically at Th17-cell-associated genes. IRF8, an alternative binding partner for BATF, negatively regulates Th17-cell differentiation (Ouyang et al., 2011). Our results identified IRF8 as a member of the Th1-cell transcriptional network whose expression was critically dependent on RA signaling. Induction of IRF8 would be expected to limit plasticity of Th1 cells by repressing Th17 differentiation, potentially by competing for binding to BATF. In support of a role for IRF8 in regulation of Th1-Th17 axis, patients with mutations in IRF8 have impaired Th1 responses (Hambleton et al., 2011) and single nucleotide polymorphisms (SNPs) in *Irf8* are associated with several autoimmune diseases in which IFN-γ^+ Th17 cells play a pathogenic role (Franke et al., 2010; Cunninghame Graham et al., 2011). It will be of interest to identify transcriptional targets of BATF, IRF4, and IRF8 in Th1 cells.

RA signaling was critical to maintain appropriate Th1-cell responses and suppress the development of IL-17^+ and IFN-γ^+IL17^+ cells. Hybrid Th1-Th17 cells are implicated in the pathogenesis of several autoimmune diseases. Their development has been attributed to the plasticity of Th17 cells. Our findings suggest that these cells might alternatively reflect Th1 plasticity and suggest a novel developmental pathway for Th17 cells. Th1 derived "Th17" cells expressed high levels of the receptor for IL-23, a critical determinant of Th17 pathogenicity (Basu et al., 2013), and were associated with significant gut inflammation and pathology in a model of oral tolerance. Further experiments are required to test the prediction that pathogenic Th17 and IFN-γ^+IL-17^+ cells which arise in autoimmunity emerge from Th1 cells when RA is deficient or its signaling perturbed.

A range of inflammatory stimuli can induce RA synthesis and signaling during the course of an immune response. Our results suggest that in a Th1-cell instructing microenvironment the dominant action of RA is to repress Th17-cell fate and promote Th1-cell responses. We did not observe enhanced Th17-cell responses during primary Th17-cell differentiation, suggesting that the impact of RA on T-cell stability might vary both temporally and among tissues. Previously we have shown in a model of skin allograft rejection that impaired Th1 responses in dn*Rara* mice were accompanied by increased Th2-cell cytokines (Pino-Lagos et al., 2011). We did not identify direct repression of Th2-cell-associated genes by RARα. However, T-bet suppresses GATA3 (Zhu et al., 2012) and in the presence of a Th2 skewing microenvironment, such as the skin, impaired expression of T-bet in the absence of RA signaling renders cells susceptible to Th2 deviation. Thus, the effects of RA on T-cell fate are likely dependent on external and intrinsic factors that shape T-cell polarity.

In summary, we show that RA signaling plays a critical role in regulating stability and functional plasticity of Th1 cells. Regulation of enhancer activity at lineage determining genes by RA-RARα provides mechanistic evidence for reciprocal regulation of Th1 and Th17-cell programs. In the absence of RA signaling, downmodulation of T-bet, STAT4, and IFN-γ, and loss of repression of Th17-cell genes, creates a permissive environment for transdifferentiation of Th1 cells to Th17 cells. This study identifies the RA-RARα axis as a potential node for intervention in diseases in which dysregulation of the Th1-Th17-cell axis is observed.

EXPERIMENTAL PROCEDURES

Mice

C57Bl/6 dn*Rara* mice have been described previously (Pino-Lagos et al., 2011). *Ifng*^{eYFP} (GREAT) mice were purchased from the Jackson Laboratory. Mice were bred and maintained at Charles River Laboratory, UK, in pathogen-free conditions. All animal experiments were conducted in accordance with the UK Animals (Scientific Procedures) Act 1986. C57Bl/6 OTII(dn*Rara*), OTII, and *Rag1*$^{-/-}$ mice were bred and maintained at the Rockefeller University specific pathogen-free animal facility.

Cell Isolation, Cell Culture, and Flow Cytometry

Sort purified, naive $CD4^+CD25^-CD44^{lo}CD62L^{hi}$ T cells were cultured with T-cell depleted splenocytes (APCs) and anti-CD3 under polarization conditions for Th0, Th1, Th2, and Th17-cell-associated subsets. Details are provided in the Supplemental Experimental Procedures. For analysis of cytokine production, cells were restimulated with 100 ng/ml phorbol 12-myristate 13-acetate (PMA) and 500 ng/ml ionomycin in the presence of monensin for 4–5 hr at 37°C. Cells were stained with LIVE/DEAD Dead Cell Stain (Invitrogen), followed by staining for cell-surface markers and then fixed and permeabilized (BD Biosciences) for staining of intracellular antigens. Flow cytometry was performed on a LSR Fortessa (BD Biosciences) and analyzed with Flowjo software (Tree Star).

TAT-Cre Transduction

Sort purified naive $CD4^+$ T cells were differentiated under Th1 conditions. After 5 days, cells were treated with 50 μg/ml TAT-Cre peptide for 45 min at 37°C, then washed and expanded in IL-2-containing medium. After 48 hr cells were retreated with Tat-Cre followed by polarization under Th1 conditions.

Real-Time Quantitative PCR

Total RNA was extracted from cells with RNeasy Mini kit (QIAGEN) and cDNA was synthesized with Qscript RT kit (Quanta). Quantitative gene-expression analysis was performed using Taqman primer probe sets (Applied Biosystems), listed in Table S4. Expression of target genes was normalized to β-actin.

Microarray Gene-Expression Profiling

For gene-expression analysis Affymetrix (for *Ifng*^{eYFP} dataset) or Agilent (for the dn*Rara* Th1 dataset) microarray chips were used. Differentially expressed genes were detected using fold-change and t test analysis. See Supplemental Experimental Procedures for further information.

Chromatin Immunoprecipitation and ChIP-Seq

Immunoprecipitation and DNA sequencing was performed by Active Motif. The following antibodies were used: anti-H3K27me3 (Millipore 07–449), anti-p300 (Santa Cruz sc–551X), anti-H3K4me1 (Active Motif 39287), anti-H3K4me3 (Active Motive 39159), anti-H3K27ac (active Motif 39133), anti-RARα (Diagenode C15310155). Illumina sequencing libraries were prepared from the ChIP and Input DNAs. For ChIP q-PCR, enrichment calculated as binding events per 1,000 cells using Active Motif's normalization scheme. Detailed methods for ChIP-seq and binding site analyses are provided in the Supplemental Information.

L. monocytogenes Infection

Mice were infected i.v. with 1×10^6 cfu *L. monocytogenes* and spleens were harvested 7 days later. Splenocytes were enriched for $CD4^+$ T cells with a $CD4^+$ T-cell negative selection microbead kit (Miltenyi Biotec) and stained with PE labeled, LLO:I-A^b dextramer (Immudex) and cell-surface antibodies. For analysis of intracellular cytokine production, splenocytes were restimulated with LLO peptide (PiProteomics) at 10 μg/ml for 6 hr.

Food-Antigen-Induced Diarrhea Model

Naive $CD4^+$ T cells from OTII or OTII(dn*Rara*) were intravenously transferred to $Rag1^{-/-}$ mice. These mice were then maintained on a diet containing OVA for 7 days and challenged with oral OVA on days 9 and 10. Lymphocytes were isolated from the intestinal epithelium, lamina propria, MLN, and spleen at the indicated time points after the start of oral OVA exposure of the recipient mice. Detailed experimental procedures are described in the Supplemental Experimental Procedures.

Statistical Analysis

Statistical significance was calculated by unpaired two-tailed Student's t test with Graphpad Prism software. p values < 0.05 were considered significant. p values are denoted in figures as follows: *, p < 0.05; **, p < 0.01; ***, p < 0.001; ****, p < 0.0001.

ACCESSION NUMBERS

Chip-seq and microarray data are available under GEO accession number GSE60356.

SUPPLEMENTAL INFORMATION

Supplemental Information includes seven figures, five tables, and Supplemental Experimental Procedures and can be found with this article online at http://dx.doi.org/10.1016/j.immuni.2015.02.003.

AUTHOR CONTRIBUTIONS

C.C.B. designed the studies, performed most of the experiments, analyzed the data, and wrote the manuscript. D.E., M.L., and D.M. performed and analyzed gut-inflammation studies. A.S., I.O.-G., and R.a.-B. assisted in processing of samples for in vitro co-culture studies and qPCR. R.E. and C.O. assisted with processing of tissues for phenotyping studies. M.A. provided advice and performed microarrays. V.P. analyzed ChIP sequencing data. E.d.R. supervised ChIP-seq data analysis. G.M.L. provided advice and supervision. D.M. designed gut-inflammation studies and contributed to the writing of the manuscript. R.J.N. supervised the overall study and contributed to the writing of the manuscript.

ACKNOWLEDGMENTS

We are indebted to PJ Chana and H. Graves (BRC Flow Cytometry Core, Kings College) for cell sorting. We gratefully acknowledge the NIH Tetramer Core Facility for provision of I-A^b:$LLO_{190-201}$ monomers and thank M. Jenkins (University of Minnesota) for providing us with Lm-2W. We thank K. Jones for critical reading of the manuscript and A. Hertweck for advice on experiments. This work was supported by a Wellcome Trust Research Training Fellowship to C.C.B. and a Wellcome Trust Principal Research Fellowship Award and NIH R01AT005382 award to R.J.N. Research was supported in part by the National Institute for Health Research (NIHR) Biomedical Research Centre, Guy's and St Thomas' National Health Service (NHS) Foundation Trust and King's College London and the Medical Research Council (MRC) Centre for Transplantation, King's College London, UK–MRC grant no. MR/J006742/1. The views expressed are those of the authors and not necessarily those of the NHS, the NIHR, or the Department of Health.

Received: August 16, 2014
Revised: December 18, 2014
Accepted: January 16, 2015
Published: March 10, 2015

REFERENCES

Annunziato, F., Cosmi, L., Santarlasci, V., Maggi, L., Liotta, F., Mazzinghi, B., Parente, E., Filì, L., Ferri, S., Frosali, F., et al. (2007). Phenotypic and functional features of human Th17 cells. J. Exp. Med. *204*, 1849–1861.

Aoyama, K., Saha, A., Tolar, J., Riddle, M.J., Veenstra, R.G., Taylor, P.A., Blomhoff, R., Panoskaltsis-Mortari, A., Klebanoff, C.A., Socié, G., et al. (2013). Inhibiting retinoic acid signaling ameliorates graft-versus-host disease by modifying T-cell differentiation and intestinal migration. Blood *122*, 2125–2134.

Basu, R., Hatton, R.D., and Weaver, C.T. (2013). The Th17 family: flexibility follows function. Immunol. Rev. *252*, 89–103.

Chanda, B., Ditadi, A., Iscove, N.N., and Keller, G. (2013). Retinoic acid signaling is essential for embryonic hematopoietic stem cell development. Cell *155*, 215–227.

Chen, C.Y., Morris, Q., and Mitchell, J.A. (2012). Enhancer identification in mouse embryonic stem cells using integrative modeling of chromatin and genomic features. BMC Genomics *13*, 152–170.

Ciofani, M., Madar, A., Galan, C., Sellars, M., Mace, K., Pauli, F., Agarwal, A., Huang, W., Parkurst, C.N., Muratet, M., et al. (2012). A validated regulatory network for Th17 cell specification. Cell *151*, 289–303.

Cunninghame Graham, D.S., Morris, D.L., Bhangale, T.R., Criswell, L.A., Syvänen, A.-C., Rönnblom, L., Behrens, T.W., Graham, R.R., and Vyse, T.J. (2011). Association of NCF2, IKZF1, IRF8, IFIH1, and TYK2 with systemic lupus erythematosus. PLoS Genet. *7*, e1002341.

Franke, A., McGovern, D.P.B., Barrett, J.C., Wang, K., Radford-Smith, G.L., Ahmad, T., Lees, C.W., Balschun, T., Lee, J., Roberts, R., et al. (2010). Genome-wide meta-analysis increases to 71 the number of confirmed Crohn's disease susceptibility loci. Nat. Genet. *42*, 1118–1125.

Hall, J.A., Cannons, J.L., Grainger, J.R., Dos Santos, L.M., Hand, T.W., Naik, S., Wohlfert, E.A., Chou, D.B., Oldenhove, G., Robinson, M., et al. (2011). Essential role for retinoic acid in the promotion of CD4(+) T cell effector responses via retinoic acid receptor alpha. Immunity *34*, 435–447.

Hambleton, S., Salem, S., Bustamante, J., Bigley, V., Boisson-Dupuis, S., Azevedo, J., Fortin, A., Haniffa, M., Ceron-Gutierrez, L., Bacon, C.M., et al. (2011). IRF8 mutations and human dendritic-cell immunodeficiency. N. Engl. J. Med. *365*, 127–138.

Hawkins, R.D., Larjo, A., Tripathi, S.K., Wagner, U., Luu, Y., Lönnberg, T., Raghav, S.K., Lee, L.K., Lund, R., Ren, B., et al. (2013). Global chromatin state analysis reveals lineage-specific enhancers during the initiation of human T helper 1 and T helper 2 cell polarization. Immunity *38*, 1271–1284.

Irvin, C., Zafar, I., Good, J., Rollins, D., Christianson, C., Gorska, M.M., Martin, R.J., and Alam, R. (2014). Increased frequency of dual-positive TH2/TH17 cells in bronchoalveolar lavage fluid characterizes a population of patients with severe asthma. J. Allergy Clin. Immunol. *134*, 1175–1186.e7.

Kamei, Y., Xu, L., Heinzel, T., Torchia, J., Kurokawa, R., Gloss, B., Lin, S.C., Heyman, R.A., Rose, D.W., Glass, C.K., and Rosenfeld, M.G. (1996). A CBP integrator complex mediates transcriptional activation and AP-1 inhibition by nuclear receptors. Cell *85*, 403–414.

Kashyap, V., Laursen, K.B., Brenet, F., Viale, A.J., Scandura, J.M., and Gudas, L.J. (2013). RARγ is essential for retinoic acid induced chromatin remodeling and transcriptional activation in embryonic stem cells. J. Cell Sci. *126*, 999–1008.

Kebir, H., Ifergan, I., Alvarez, J.I., Bernard, M., Poirier, J., Arbour, N., Duquette, P., and Prat, A. (2009). Preferential recruitment of interferon-gamma-expressing TH17 cells in multiple sclerosis. Ann. Neurol. *66*, 390–402.

Klebanoff, C.A., Spencer, S.P., Torabi-Parizi, P., Grainger, J.R., Roychoudhuri, R., Ji, Y., Sukumar, M., Muranski, P., Scott, C.D., Hall, J.A., et al. (2013). Retinoic acid controls the homeostasis of pre-cDC-derived splenic and intestinal dendritic cells. J. Exp. Med. *210*, 1961–1976.

Komatsu, N., Okamoto, K., Sawa, S., Nakashima, T., Oh-hora, M., Kodama, T., Tanaka, S., Bluestone, J.A., and Takayanagi, H. (2014). Pathogenic conversion of Foxp3+ T cells into TH17 cells in autoimmune arthritis. Nat. Med. *20*, 62–68.

Mucida, D., Park, Y., Kim, G., Turovskaya, O., Scott, I., Kronenberg, M., and Cheroutre, H. (2007). Reciprocal TH17 and regulatory T cell differentiation mediated by retinoic acid. Science *317*, 256–260.

Mukasa, R., Balasubramani, A., Lee, Y.K., Whitley, S.K., Weaver, B.T., Shibata, Y., Crawford, G.E., Hatton, R.D., and Weaver, C.T. (2010). Epigenetic instability of cytokine and transcription factor gene loci underlies plasticity of the T helper 17 cell lineage. Immunity *32*, 616–627.

Nistala, K., Adams, S., Cambrook, H., Ursu, S., Olivito, B., de Jager, W., Evans, J.G., Cimaz, R., Bajaj-Elliott, M., and Wedderburn, L.R. (2010). Th17 plasticity in human autoimmune arthritis is driven by the inflammatory environment. Proc. Natl. Acad. Sci. USA *107*, 14751–14756.

Ouyang, X., Zhang, R., Yang, J., Li, Q., Qin, L., Zhu, C., Liu, J., Ning, H., Shin, M.S., Gupta, M., et al. (2011). Transcription factor IRF8 directs a silencing programme for TH17 cell differentiation. Nat. Commun. *2*, 314.

Pino-Lagos, K., Guo, Y., Brown, C., Alexander, M.P., Elgueta, R., Bennett, K.A., De Vries, V., Nowak, E., Blomhoff, R., Sockanathan, S., et al. (2011). A retinoic acid-dependent checkpoint in the development of CD4+ T cell-mediated immunity. J. Exp. Med. *208*, 1767–1775.

Rada-Iglesias, A., Bajpai, R., Swigut, T., Brugmann, S.A., Flynn, R.A., and Wysocka, J. (2011). A unique chromatin signature uncovers early developmental enhancers in humans. Nature *470*, 279–283.

Reis, B.S., Rogoz, A., Costa-Pinto, F.A., Taniuchi, I., and Mucida, D. (2013). Mutual expression of the transcription factors Runx3 and ThPOK regulates intestinal $CD4^+$ T cell immunity. Nat. Immunol. *14*, 271–280.

Schraml, B.U., Hildner, K., Ise, W., Lee, W.-L., Smith, W.A.E., Solomon, B., Sahota, G., Sim, J., Mukasa, R., Cemerski, S., et al. (2009). The AP-1 transcription factor Batf controls T(H)17 differentiation. Nature *460*, 405–409.

Schulz, E.G., Mariani, L., Radbruch, A., and Höfer, T. (2009). Sequential polarization and imprinting of type 1 T helper lymphocytes by interferon-gamma and interleukin-12. Immunity *30*, 673–683.

Spencer, S.P., Wilhelm, C., Yang, Q., Hall, J.A., Bouladoux, N., Boyd, A., Nutman, T.B., Urban, J.F., Jr., Wang, J., Ramalingam, T.R., et al. (2014). Adaptation of innate lymphoid cells to a micronutrient deficiency promotes type 2 barrier immunity. Science *343*, 432–437.

Takahashi, H., Kanno, T., Nakayamada, S., Hirahara, K., Sciumè, G., Muljo, S.A., Kuchen, S., Casellas, R., Wei, L., Kanno, Y., and O'Shea, J.J. (2012). TGF-β and retinoic acid induce the microRNA miR-10a, which targets Bcl-6 and constrains the plasticity of helper T cells. Nat. Immunol. *13*, 587–595.

Urvalek, A.M., and Gudas, L.J. (2014). Retinoic acid and histone deacetylases regulate epigenetic changes in embryonic stem cells. J. Biol. Chem. *289*, 19519–19530.

Vahedi, G., Takahashi, H., Nakayamada, S., Sun, H.-W., Sartorelli, V., Kanno, Y., and O'Shea, J.J. (2012). STATs shape the active enhancer landscape of T cell populations. Cell *151*, 981–993.

Wadia, J.S., Stan, R.V., and Dowdy, S.F. (2004). Transducible TAT-HA fusogenic peptide enhances escape of TAT-fusion proteins after lipid raft macropinocytosis. Nat. Med. *10*, 310–315.

Wei, L., Vahedi, G., Sun, H.-W., Watford, W.T., Takatori, H., Ramos, H.L., Takahashi, H., Liang, J., Gutierrez-Cruz, G., Zang, C., et al. (2010). Discrete roles of STAT4 and STAT6 transcription factors in tuning epigenetic modifications and transcription during T helper cell differentiation. Immunity *32*, 840–851.

Xiao, S., Jin, H., Korn, T., Liu, S.M., Oukka, M., Lim, B., and Kuchroo, V.K. (2008). Retinoic acid increases Foxp3+ regulatory T cells and inhibits development of Th17 cells by enhancing TGF-beta-driven Smad3 signaling and inhibiting IL-6 and IL-23 receptor expression. J. Immunol. *181*, 2277–2284.

Yang, Y., Ochando, J.C., Bromberg, J.S., and Ding, Y. (2007). Identification of a distant T-bet enhancer responsive to IL-12/Stat4 and IFNgamma/Stat1 signals. Blood *110*, 2494–2500.

Zhang, F., Meng, G., and Strober, W. (2008). Interactions among the transcription factors Runx1, RORgammat and Foxp3 regulate the differentiation of interleukin 17-producing T cells. Nat. Immunol. *9*, 1297–1306.

Zhou, L., Ivanov, I.I., Spolski, R., Min, R., Shenderov, K., Egawa, T., Levy, D.E., Leonard, W.J., and Littman, D.R. (2007). IL-6 programs T(H)-17 cell differentiation by promoting sequential engagement of the IL-21 and IL-23 pathways. Nat. Immunol. *8*, 967–974.

Zhu, J., Jankovic, D., Oler, A.J., Wei, G., Sharma, S., Hu, G., Guo, L., Yagi, R., Yamane, H., Punkosdy, G., et al. (2012). The transcription factor T-bet is induced by multiple pathways and prevents an endogenous Th2 cell program during Th1 cell responses. Immunity *37*, 660–673.

Immunity
Supplemental Information

Retinoic Acid Is Essential for Th1 Cell Lineage Stability and Prevents Transition to a Th17 Cell Program

Chrysothemis C. Brown, Daria Esterhazy, Aurelien Sarde, Mariya London, Venu Pullabhatla, Ines Osma-Garcia, Raya al-Bader, Carla Ortiz, Raul Elgueta, Matthew Arno, Emanuele de Rinaldis, Daniel Mucida, Graham M. Lord, and Randolph J. Noelle

Figure S1 (related to Figure 1). Expression of Foxp3 in CD4$^+$ T-cells deficient in RA signaling

(A) Intracellular expression of Foxp3 in CD4$^+$ T-cells from spleen, thymus and mesenteric lymph nodes (MLN) of wild-type littermate control (WT) and dn*Rara* mice.

(B) Total number of CD4$^+$Foxp3$^+$ T-cells in spleen (upper panel) and thymus (lower panel) of WT and dn*Rara* mice

Data are representative of two independent experiments. Mean ± SEM.

Figure S1.

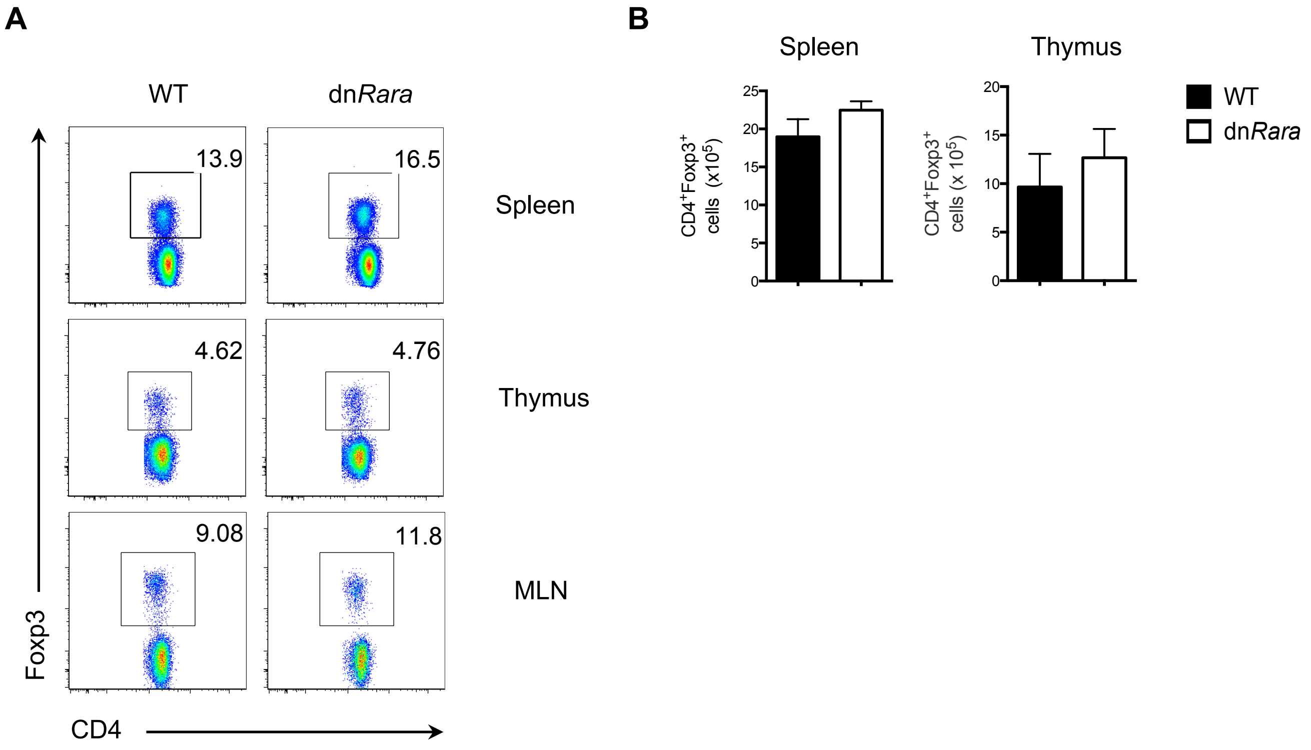

A
WT
dnRara
13.9
16.5
Spleen
4.62
4.76
Thymus
9.08
11.8
MLN
Foxp3
CD4
B
Spleen
Thymus
CD4+Foxp3+ cells (x10^5)
25
20
15
10
5
0
WT
dnRara

Figure S2 (related to Figure 2). Proliferation and differentiation of CD4$^+$ T-cells in the absence of RA signaling

(A) Naïve CD4+ T-cells from WT and dn*Rara* mice were labeled with CellTrace^{TM} and cultured under Th1 conditions for 5 days. Flow cytometry showing dye dilution, gated on viable CD4$^+$ T-cells.

(B) Cell-surface expression of CD44 and CD25 on naïve CD4+ T-cells from WT or dn*Rara* mice cultured under Th1 conditions for 5 days.

(C) Naïve CD4+ T-cell from WT and dn*Rara* mice were cultured under Th0 or Th2 conditions for 6 days. Cells were analysed by flow cytometry for expression of intracellular RORγt. Gated on CD4$^+$ T-cells.

(D) Sorted naïve CD4$^+$ T- cells from WT and dn*Rara* mice were cultured under Th17 conditions for 6 days. Intracellular IL-17A and IFN-γ expression after stimulation with PMA and ionomycin.

(E) CD4$^+$ T-cells from dn*Rara-Ifng*^{eYFP} and *Ifng*^{eYFP} mice were cultured under Th1 conditions. Quantitative real-time PCR analysis of *Cxcr3* and *Il12rb2* from IFN-γ$^+$ (eYFP$^+$) cells sorted on day 7. Samples from three independent experiments.

Representative data from two to three independent experiments (A-D). Mean ± SEM.

Figure S2.

A

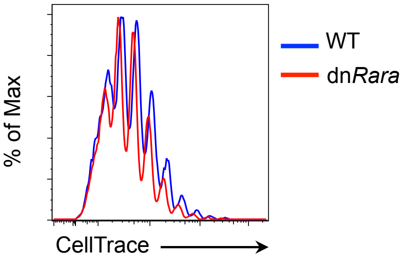

	WT	dn*Rara*
% cells divided	90.83	92.67
Proliferation index	3.65	3.86

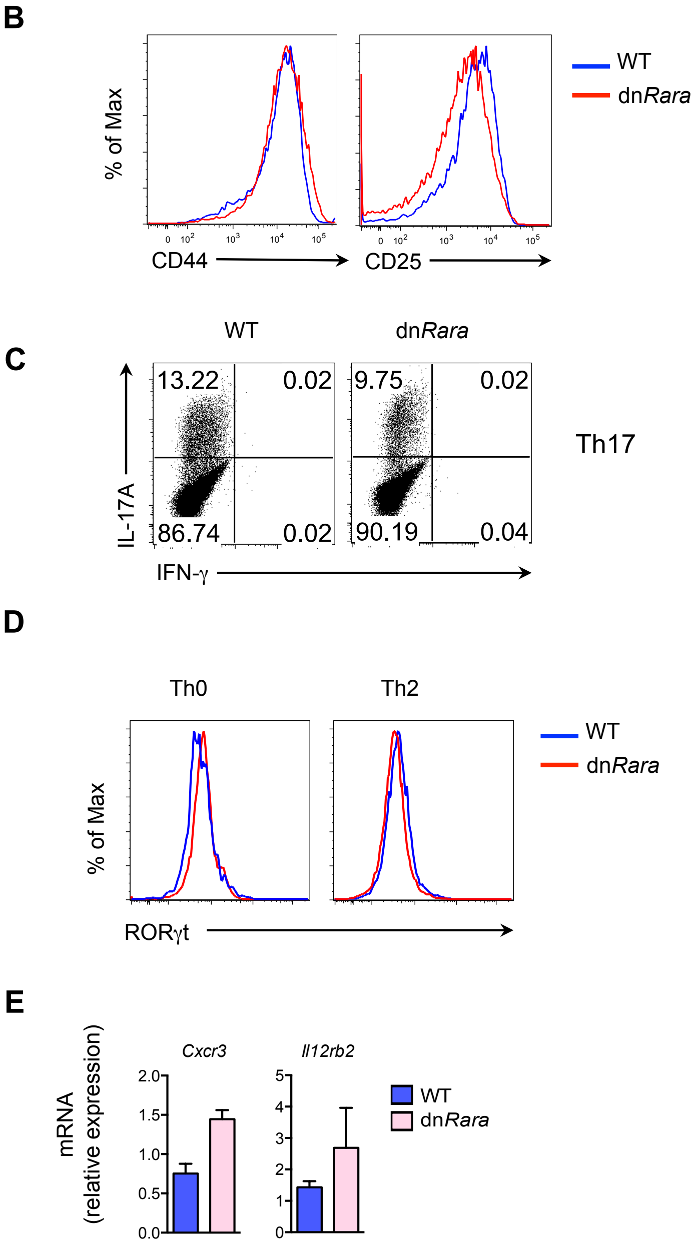

Figure S3 (related to Figure 3). STAT3 and STAT4 activity in dn*Rar*a Th1 differentiated cells

(A) Flow cytometric analysis of STAT3 and STAT4 phosphorylation in naïve $CD4^+$ T-cells from dn*Rara* and WT mice differentiated under Th1 conditions. Cells analysed after 6 days following treatment with 25ng/ml IL-12, 20ng/ml IL-6 and 10ng/ml IL-23 for 30 minutes. Dashed lines represent untreated cells.

(B) Bar graph depicts ratio of pSTAT3/pSTAT4 signaling as assessed by MFI.

Figure S3.

A

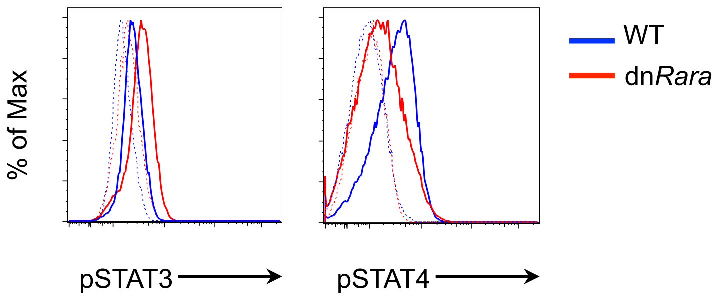

WT
dn*Rara*
% of Max
pSTAT3
pSTAT4

B

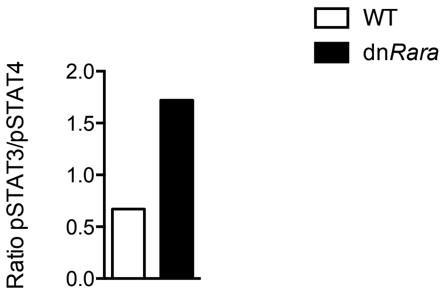

WT
dn*Rara*
Ratio pSTAT3/pSTAT4
2.0
1.5
1.0
0.5
0.0

Figure S4 (related to Figure 4). Cytokine analysis following temporal inhibition of RA signaling in Th1 cells

(A) Naive $CD4^+$ T-cells from dn*Rara*$^{lsl/lsl}$ mice were cultured under Th1 conditions. Th1 cells were transduced with TAT-Cre on days 5 and 7 and repolarised under Th1 conditions for a further 5 days. Intracellular expression of IFN-γ and IL-17A following PMA and ionomycin stimulation.

(B) Naive CD4+ T-cells from *Ifng*^{eYFP} mice were differentiated under Th1 conditions. IFN-γ^+ ($eYFP^+$) cells were sorted on day 7 and recovered cells underwent secondary repolarisation in Th1 conditions for 5 days in the presence of Veh or RAi. Intracellular expression of IFN-γ and IL-17A following PMA and ionomycin stimulation.

Data representative of two independent experiments.

Figure S4.

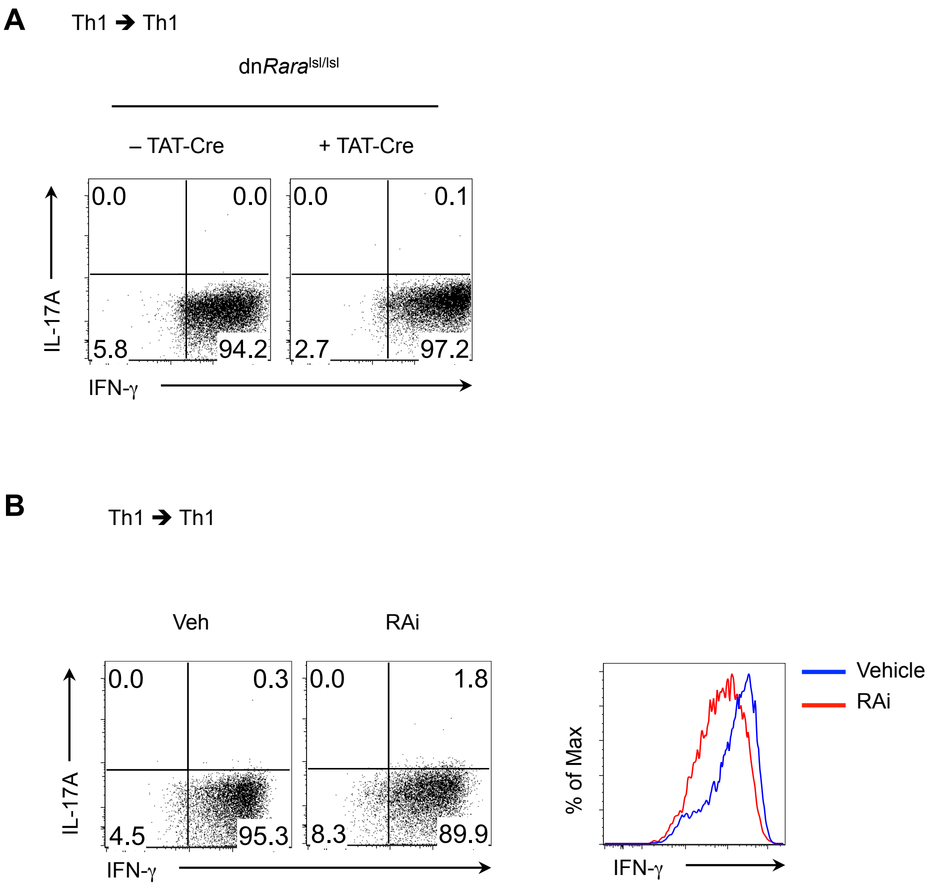
A
Th1 ➔ Th1
dnRara lsl/lsl
– TAT-Cre
+ TAT-Cre
0.0
0.0
5.8
94.2
0.0
0.1
2.7
97.2
IL-17A
IFN-γ
B
Th1 ➔ Th1
Veh
RAi
0.0
0.3
4.5
95.3
0.0
1.8
8.3
89.9
IL-17A
IFN-γ
% of Max
IFN-γ
Vehicle
RAi

Figure S5 (related to Figure 5). RA-RARα regulates enhancers at Th1 genes and represses Th17 lineage specifying genes

Naive $CD4^+$ T-cells from dn*Rara* and WT mice were cultured under Th1 conditions as in Figure 5. After 6 days, ChIP was performed with the specified antibodies, followed by real-time PCR analysis at selected sites (B-C) or sequencing (A).

(A) ChIP-seq binding tracks at *Stat4* and *Ifng* loci for RARα in WT Th1 polarised cells and p300 binding, H3K27ac, H3K4me1 and H3K4me3 modifications in WT and dn*Rara* Th1 cells.

(B) Validation of the RARα ChIP-seq regions in (A) by ChIP-qPCR assays. Untr6 region serves as a negative control. Data presented normalised to input.

(C) ChIP analysis of the abundance of p300 at the loci in (B) in WT and dn*Rara* Th1 cells. Data presented normalised to input.

(D) ChIP-seq analysis of STAT4 binding at the Tbx21 enhancer and comparison of p300 binding in WT and $STAT4^{-/-}$ Th1 cells. ChIP-Seq data (Vahedi et al. 2012 and Wei et al., 2010) was mapped to the Dec. 2011 (GRCm38/mm10) mouse genome assembly with the UCSC genome browser along with the ChIP-seq binding track for RARα at the *Tbx21* locus.

(E) Quantitative real time PCR analysis of selected genes identified as differentially expressed on genome wide transcriptional profiling analysis of cells as in (A). Mean ± SEM.

(F) Cell-surface expression of IL6-Rα by flow cytometry in naïve dn*Rara* and WT $CD4^+$ T-cells at indicated timepoints. Grey histogram indicates staining for isotype control.

Data (B-F) representative of two to three independent experiments. Mean ± SD unless otherwise stated, $^{**}p < 0.01$; $^{****}p < 0.0001$.

Figure S5.

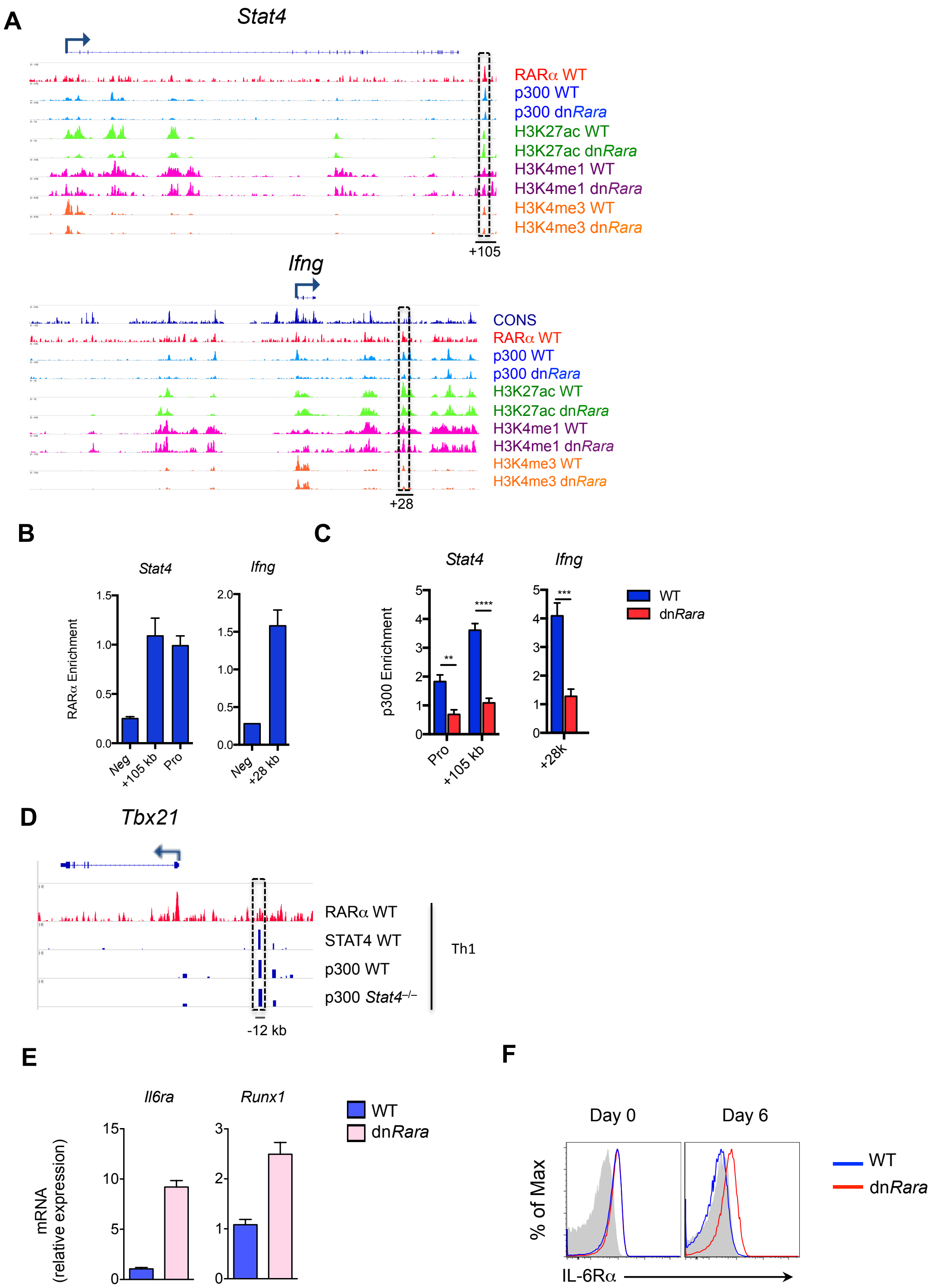

A
Stat4
RARα WT
p300 WT
p300 dnRara
H3K27ac WT
H3K27ac dnRara
H3K4me1 WT
H3K4me1 dnRara
H3K4me3 WT
H3K4me3 dnRara
+105
Ifng
CONS
RARα WT
p300 WT
p300 dnRara
H3K27ac WT
H3K27ac dnRara
H3K4me1 WT
H3K4me1 dnRara
H3K4me3 WT
H3K4me3 dnRara
+28
B
Stat4
Ifng
RARα Enrichment
Neg
+105 kb
Pro
Neg
+28 kb
C
Stat4
Ifng
p300 Enrichment
WT
dnRara
Pro
+105 kb
+28k
D
Tbx21
RARα WT
STAT4 WT
p300 WT
p300 Stat4−/−
Th1
-12 kb
E
Il6ra
Runx1
mRNA (relative expression)
WT
dnRara
F
Day 0
Day 6
% of Max
IL-6Rα
WT
dnRara

Figure S6 (related to Figure 6). Cytokine production by dnRARa T-cells following infection with L. monocytogenes

(A) Splenocytes from dn*Rara* and WT mice infected with Lm-2W were restimulated with LLOp for 24 h. Concentration of IFN-γ, IL-17A and IL-4 in supernatants was measured by multiplex bead array (Biorad). Data normalised to total numbers of $CD4^+$ T-cells. n = 3-4 mice per group.

(B) Intracellular staining for IFN-γ and IL-4 following stimulation of splenocytes with LLOp for 6 h, 7 days after infection with *L. monocytogenes*. Gated on $CD3^+CD4^+$ T-cells

(C) Cell surface expression of IL-6Rα by flow cytometry on LLOp:I-A^b CD4+ T-cells isolated from spleen of dn*Rara* or WT mice 7 days after infection with *L. monocytogenes*. Data from 4 pooled mice. Numbers indicate MFI.

Data representative of two to three independent experiments. Mean ± SEM.

Figure S6.

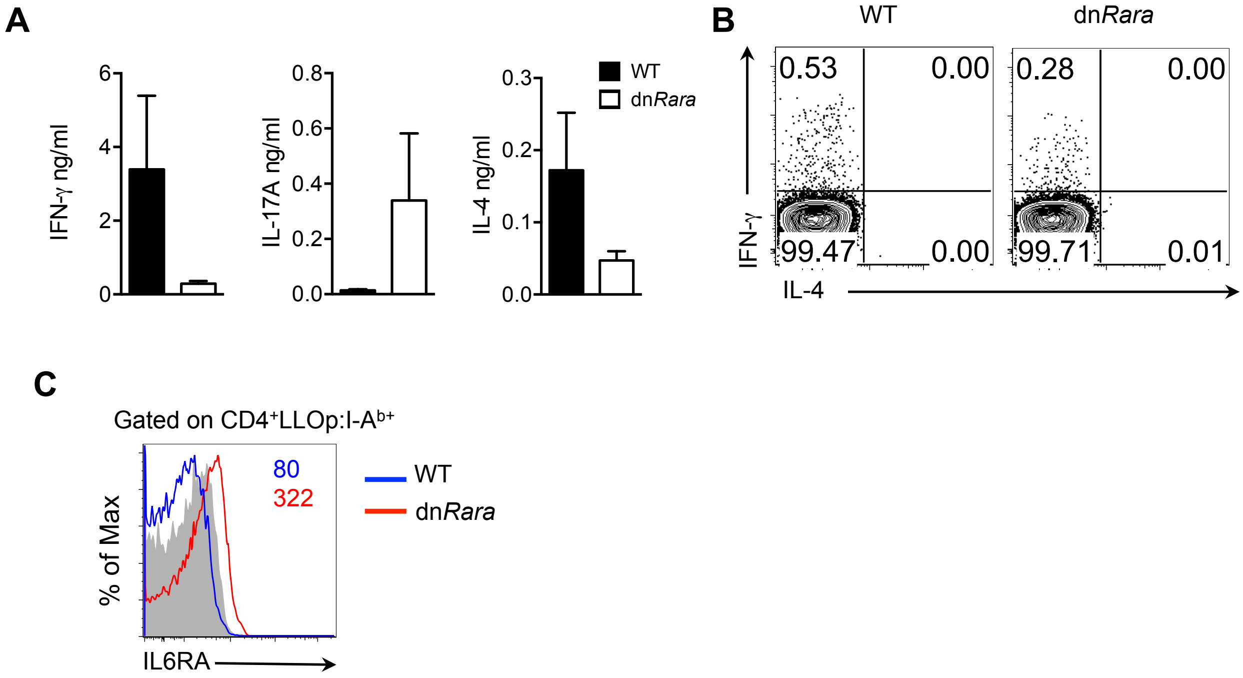

A
IFN-γ ng/ml
6
4
2
0
IL-17A ng/ml
0.8
0.6
0.4
0.2
0.0
IL-4 ng/ml
0.3
0.2
0.1
0.0
WT
dn*Rara*
B
WT
dn*Rara*
0.53
0.00
99.47
0.00
0.28
0.00
99.71
0.01
IFN-γ
IL-4
C
Gated on CD4⁺LLOp:I-Ab⁺
80
322
% of Max
IL6RA
WT
dn*Rara*

Figure S7 (related to Figure 7). Gut homing in dn*Rara*-OTII CD4+ T-cells
(A) Percentage of OTII or OTII(dn*Rara*) $CD4^+$ cells recovered from LPL, IEL, MLN and Spleen of $RAG^{-/-}$ recipients, 9 days after adoptive transfer (n = 3-4 per group). Data representative of two independent experiments. Mean ± SEM.

Figure S7.

A

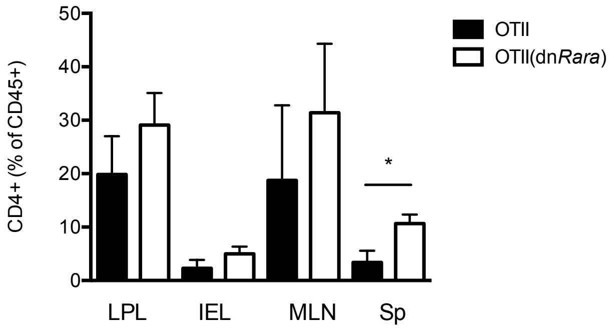

Table S1 (related to Figure 5). List of Sequencing-Based Data Used in This Study including publically available data as indicated by Geo Accession Number

Samples	Non-redundant tags	Peak counts
RARA_WT	13303876	1776
H3K4me1_DNRAR	18605274	65960
H3K4me1_WT	23760603	49542
H3K4me3_DNRAR	18333386	49505
H3K4me3_WT	21918629	53135
H3K27Ac_DNRAR	17421600	37788
H3K27Ac_WT	20513640	37151
H3K27me3_DNRAR	30667883	56002
H3K27me3_WT	20833021	78511
p300_DNRAR	23023765	30495
p300_WT	25213927	46191
Stat4 WTTh1 (GSM550303)	8982352	20862
p300 WT Th1 (GSM994508)	19652779	25554
p300 Stat4$^{-/-}$ Th1 (GSM994509)	18282554	29208

Table S2 (related to Figure 5). Genes downregulated in dn*Rara* Th1 cells that were bound by RARα in WT Th1 cells.

1110037F02Rik	Fli1	Ncln
1810011H11Rik	Fmnl3	Nedd4l
3300005D01Rik	Foxo3	Nfic
5830416P10Rik	Foxp1	Nln
Acsl4	Furin	Nme1
Adora2a	Gas5	Nod1
Alkbh7	Gcsh	Notch2
Asb2	Gfi1	Nt5e
Birc5	Gimap3	P2rx7
Blm	Gimap4	Pde2a
Bre	Gimap8	Prr5l
Capzb	Gimap9	Rbks
Chsy1	Hic1	Rcbtb2
Cmas	Hmgcs1	Shf
Cnga1	Idi1	Slc16a6
Coq7	Ifngr1	Smad3
Ctps	Ifrd2	Sqle
Cycs	Irf8	Sulf2

Cyp51	Itih5	Tbx21
Cyp51	Kcnn4	Treml2
Dennd4a	Kif2c	Txn2
Dusp6	Lbr	Ube2e3
E2f3	Lef1	Uchl3
Enpp4	Mdc1	Vav3
Fasn	Me2	Vipr1
Fgl2	Mrto4	

Table S3 (related to Figure 5). Genes upregulated in dn*Rara* Th1 cells that were bound by RARα in WT Th1 cells

1110038F14Rik	Ifngr2	Slfn2
Ak2	Il15ra	Socs1
Antxr2	Insr	Sp100
Aph1b	Irf1	Stat1
Arhgap25	Irgm1	Tagap
Arid4a	Kif3b	Tmem50a
B2m	Mcl1	Tnip1
Bace2	Mettl8	Tor1aip2
Bcl10	Mga	Traf1
Bcl6	Mpeg1	Trpm6
Birc3	Nek6	Twsg1
Cd320	Net1	Usp53
Cnnm2	Npc2	Vav1
Ddit3	Plec	Wdsub1
Egr2	Polg	Zbp1
Fam43a	Ptpn1	Zfp207
Filip1l	Rab19	Zfp36l2
Fndc3a	Rhd	Zmym6
Fuca1	Slamf1	

Table S4. Taqman assays used for RT-PCR gene expression analyses (related to Figures 1-3 and 5).

Mouse ACTB	4352341E
Il6ra	Mm00439653_m1
Il22	Mm00444241_m1
Runx1	Mm01213404_m1
Batf	Mm00479410_m1
Cxcr3	Mm99999054_s1

Il23r	Mm00519943_m1
Il1r1	Mm00434237_m1
Il21	Mm00517640_m1
Il10	Mm00439616_m1
Irf8	Mm00492567_m1
Irf4	Mm00516431_m1
Stat4	Mm00448890_m1
Il12rb2	Mm00434200_m1
Ifng	Mm00801778_m1
Il12rb1	Mm00434189_m1
Rorc	Mm01261022_m1
Gata3	Mm00484683_m1
Tbx21	Mm00450960_m1

Table S5 (related to Figure 5). Sequences of PCR primers used in ChIP assays

Stat4_+105k F	TCCTCCTCCCTTTGTTGTTC
Stat4 +105k R	GGGCCTTAATCAACCATTTC
Stat4 Promoter F	AGAGGGCATACACCGAGAAC
Stat4 Promoter R	TCTAGGGAGCCAGCATCAAC
Tbx21 Promoter F	TCGCTTTTGGTGAGGACTG
Tbx21 Promoter R	GGTGGCAGGTTGACTCTTTC
Tbx21 -12k F	GCGGAAGAGGGAACTAACAC
Tbx21 -12k R	GGACCCGGAACCTATGTATG
Irf8 Promoter F	CAGAAGCTAGGGCTGGTGTC
Irf8 Promoter R	CACAGAACAGATCCCAAATGTC
Irf8 -11k F	CCTTAACCCCGGAACTGTAG
Irf8 -11k R	TGCTGTGCTTGCCTCTACTC
Il6ra Promoter F	TCCGCTTGAGTTTTGCTTTC
Il6ra Promoter R	CACTGACCTGCCTTCTACTTTAAC
Il6ra +32k F	CAAAGCTAAAACCAGGAAATGAC
Il6ra +32k R	AAAAGGTTCCATGTGATGTTG
Rorc Promoter (Rorγt isoform) F	AGGAATTTGGGTGTGGTGAG
Rorc Promoter (Rorγt isoform) R	CTGTCTTGGGTGGTGTCTTG
Runx1 Promoter 1 F	TGGAAGAGGAAGAAGCTGTG
Runx1 Promoter 1 R	CAAGAGAAGCCACCCCAAAC
Runx1 Promoter 2 F	TGCTGGGCTTACACTTCTGAC
Runx1 Promoter 2 R	TGGACCTCATAAACAACCACAG
IFNg +28k F	CTTTGAGCCACTGATGGGTAG
IFNg +28k R	GCCTCTCCACGTCTCTTCTTC

Supplemental Experimental Procedures

Reagents

$LLO_{190-201}$ was synthesised by PiProteomics and was >95% pure, as determined by HPLC. LLO:I-A^b monomers were provided by NIH Core Tetramer Facility. PE labeled LLO:I-A^b dextramers were synthesised by Immudex. Recombinant Lm-2W strain was provided by Marc Jenkin's Laboratory. LE540 was purchased from Alpha Laboratories.

Naïve CD4+ T-cell isolation and culture

Naïve $CD4^+CD25^{neg}CD44^{lo}CD62L^{hi}$ T-cells were isolated by cell sorting by FACSAria (BD) after enrichment with a $CD4^+$ T-cell negative selection kit (Miltenyi Biotec). T-cell depleted splenocytes were prepared using a $CD3^+$ microbead selection kit (Miltenyi Biotec) followed by irradiation at 3000 rad. Naïve CD4+ T-cells were cultured for 3 days with irradiated T cell-depleted splenocytes at a ratio of 1:5 in the presence of 5 μg/ml of anti-CD3 (145-2C11) under Th0 cell conditions (IL-2 100 IU/ml, anti-IL-4 (11B11) and anti-IFN-g (XMG1.2), 10 μg/ml each); Th1 cell conditions (100 IU/ml of IL-2, 10 ng/ml of IL-12, and anti-IL-4); Th2 cell conditions (100 IU/ml of IL-2, 10 ng/ml of IL-4, anti-IL-12 (C17.8), and anti-IFN-γ (XMG1.2); or Th17 cell conditions, 5 ng/ml TGFβ, 20 ng/ml IL-6, 10 ng/ml IL-1β, anti-IL-4, and anti-IFN-γ). Cells were expanded for an additional 3-4 days. Where indicated, 10 ng/ml IFN-γ or 10 μg/ml anti-IFN-γ was added. In secondary repolarisation assays, where specified, LE540 (1 μM) or DMSO (vehicle control) was added to the media. Cytokines were from R&D. Anti-CD3 was from BioXcell and other antibodies were from BD Biosciences. All cell cultures were performed in complete RPMI containing 10% fetal

bovine serum (FBS), 55 μM β-mercaptoethanol, HEPES, non-essential amino acids, glutamine, penicillin and streptomycin.

TAT-Cre transduction

Sort purified naïve CD4+ T-cells were differentiated under Th1 conditions. After 5 days, cells were washed twice in serum free medium prior to treatment with 50 μg/ml TAT-Cre (Millipore) or medium alone (mock treatment). Cells were incubated at 37°C for 45 minutes. The reaction was quenched with medium containing 20% FBS followed by further washing. Cells were expanded for 2 days followed by retreatment with TAT-Cre or media as before. Cells were then restimulated under Th1 cell conditions for 3 days and expanded for a further 2 days prior to analysis.

Flow Cytometry

For analysis of cytokine production, cells were restimulated with 100 ng/ml phorbol 12-myristate 13-acetate (PMA) and 500 ng/ml ionomycin in the presence of monensin for 4-5 h at 37°C in a tissue culture incubator. Cell surface staining was carried out in PBS with 2% FBS. For live cell analysis or cell sorting, dead cells were excluded by staining with SYTOX blue (Invitrogen). For intracellular staining, cells were first stained with LIVE/DEAD Fixable Violet or near IR Dead Cell Stain (Invitrogen), followed by staining for cell-surface markers and then resuspended in fixation/permeabilisation solution (Cytofix/Cytoperm kit or Transcription Factor Buffer kit; BD Bioscences). Intracellular staining carried out in accordance with the manufacturer's instructions. Intracellular phosphorylated STAT proteins were stained with Phosflow Lyse/Fix Buffer, and Phosflow Perm Buffer III (BD

Biosciences) according to the manufacturer's protocol. Data were collected with a LSR Fortessa (BD) and results were analyzed with FlowJo software (Tree Star). All the antibodies for staining cell surface markers, cytokines or transcription factors were purchased from either BD Biosciences or eBiosciences.

Luminex Immunoassays

Cytokine levels in supernatants were measured using a multiplex bead-based assay (Bio-Rad Laboratories) in a Luminex FlexMap3D System (Luminex Corporation).

Western Blotting

Differentiated Th1 cells were lysed in RIPA buffer supplemented with protease inhibitors. Lysates were electrophoresed on 10% gels (Biorad), transferred to nitrocellulose and blotted with anti-STAT4 or anti-actin followed by anti-rabbit-horseradish peroxidase conjugated antibody. All antibodies were from Cell Signaling Technology.

***L. monocytogenes* infection and analysis**

Mice were infected i.v. with 1 x 10^6 cfu L. monocytogenes and spleens were harvested 7 days later. For FACS analysis, single cell suspensions were enriched for $CD4^+$ T-cells with a $CD4^+$ T-cell negative selection microbead kit (Miltenyi Biotec) and stained with PE labeled, LLO:I-A^b dextramer (Immudex) and cell surface antibodies. For analysis of cytokine production, supernatants were collected from splenocytes restimulated with LLO peptide (PiProteomics) at 10 μg/ml for 24 h or intracellular cytokine staining was performed following stimulation with LLO peptide for 6 h in the presence of monensin.

Food antigen induced diarrhoea model

Rag1$^{-/-}$ mice were kept on a sulfatrim-containing diet and only exposed to autoclaved supplies. Naïve OTII CD4 cells (defined as $CD4^{+}CD25^{-}Vb5^{+}Va2^{+}CD44^{-}$) were sorted from 8-12 weeks old female C57Bl6 OTII(dn*Rara*) or C57Bl6 OTII mice using a FACS Aria cell sorter (Becton Dickinson), and 2 x 10^{6} cells in 100µl PBS were retro-orbitally transferred to 12 weeks old Rag1$^{-/-}$ females. 12h after the adoptive transfer, the drinking water was replaced by a 1% Grade II ovalbumin (OVA, Sigma) and 0.5% Splenda (McNeil Nutritionals) solution for 7 days. Body weight was measured at 5pm every day. For monitoring diarrhea development, the faeces texture after 7 days of OVA, 2h after a gavage challenge with 50mg Grade III OVA (Sigma) in 200 µl PBS on days 9 and 10 and without further challenge on day 12 was analysed. A mouse was diagnosed with diarrhoea if the faeces had the characteristic soft and light appearance at two consecutive occasions. For the single gavage challenge experiment, mice were subjected to the challenge on day 9 only and the faeces were analysed after 2h. To determine T cell frequencies, lymphocytes were isolated as previously described (Mucida et al., 2007) on day 7 (from mesenteric lymph node (MLN) and spleen only) or day 9 (from the intestinal epithelium, lamina propria, MLN and spleen) after the start of oral OVA exposure of the recipient mice. For cytokine staining, isolated lymphocytes were stimulated for 3h in RPMI medium supplemented with 10% FBS, 55 µM β-mercaptoethanol, 100ng/ml PMA (Sigma), 500ng/ml Ionomycin (Sigma) and 10µg/ml brefeldin A (Sigma) prior to the incubation with antibodies. Cells were first stained with antibodies against cell surface markers, followed by permeabilization using either Fix/Perm buffer (BD Pharmingen) for cytokine stainings, or

using the Foxp3 Mouse Regulatory T cell Staining Kit (eBioscience) for Foxp3 staining. The fluorescent-dye- conjugated antibodies used were obtained from BD-Pharmingen (anti-CD4, 550954; anti-CD25, 553866; anti-IL-17a, 559502; anti-Vb5, 553190) or eBioscience (anti-CD44, 56-0441; anti-CD45.2, 47-0454; anti-TCR-β, 47-5961; anti-IFN-γ, 25-7311; anti-Foxp3, 17-5773; anti-Vα2, 48-5812). Stained cells were analysed using a LSR-II flow cytometer (Becton Dickinson) and population frequencies were determined using the FlowJo software (Tree Star).

Chromatin immunoprecipitation (ChIP)

20-60 million Th1 polarised cells from WT and dnRara mice were fixed, washed and snap-frozen according to the Cell Fixation protocol from Active Motif (http://www.activemotif.com/documents/1848.pdf). Chromatin was isolated by the addition of lysis buffer, followed by disruption with a Dounce homogenizer. Lysates were sonicated and the DNA sheared to an average length of 300-500 bp. Genomic DNA (Input) was prepared by treating aliquots of chromatin with RNase, proteinase K and heat for de-crosslinking, followed by ethanol precipitation. Pellets were resuspended and the resulting DNA was quantified on a NanoDrop spectrophotometer. Extrapolation to the original chromatin volume allowed quantitation of the total chromatin yield. An aliquot of chromatin was precleared with protein A agarose beads (Invitrogen). Following immunoprecipitation with specified antibodies, complexes were washed, eluted from the beads with SDS buffer, and subjected to RNase and proteinase K treatment. Crosslinks were reversed by incubation overnight at 65 C, and ChIP DNA was purified by phenol-chloroform extraction

and ethanol precipitation and used for the preparation of Illumina sequencing libraries and for ChIP qPCR analysis.

ChIP-qPCR

Quantitative PCR (qPCR) reactions were carried out in triplicate on specific genomic regions using SYBR Green Supermix (Bio-Rad). See Table S5 for Primer details. The resulting signals were normalized for primer efficiency by carrying out qPCR for each primer pair using Input DNA. By using standards of known quantities of DNA it was possible to calculate the number of genome copies pulled down for each of the sites tested, and thus to calculate the copies pulled down per starting cell number, presented as 'Enrichment'. For RARα ChIP qPCR a gene desert on chromosome 6 (Untr6) was used for a negative control site (Active Motif Catalog No: 71011).

<u>ChIP Sequencing (Illumina)</u>

Illumina sequencing libraries were prepared from the ChIP and Input DNAs using standard procedures and libraries were sequenced on HiSeq 2500

ChipSeq Analysis

For each sample the 50bp SE reads in FastQ format from the sequencer were aligned to the mouse reference genome (mm10) using Novoalign v2.07.11 (http://www.novocraft.com). The resulting alignment file was converted to BAM format using samtools (http://samtools.sourceforge.net/) and the pcr duplicates were removed using picard tools (http://picard.sourceforge.net). Only uniquely mapped reads from each

sample were selected for further analysis. Significantly enriched regions from each sample were identified with MACS v2.0.10_20131216 (Zhang et al. 2008, Feng J et al. 2011) (with q=0.10) using the input sample for background correction. In some instances peaks were identified by visual inspection and confirmed by ChIP qPCR. In case of H3K4me1 and H3K27me3 samples, "--broad" setting was used to merge nearby enriched regions. For visualization purposes, the input signal was subtracted from each ChIP sample and was converted into bigWig format using "bedGraphToBigWig" utility from UCSC tools (http://genome.ucsc.edu/util.html). The identified significantly enriched regions were annotated to find the associated genes using "FindNeighbouringGenes" utility from USeq package (http://useq.sourceforge.net/). Associated genes represent the closest transcriptional start site from the centre of the peak.

Microarray data

Total RNA was extracted from cells lysed in Trizol LS reagent (Life Technologies). RNA quality was assessed with an Agilent 2100 Bioanalyzer (Agilent Technologies) and quantified with the Nanodrop ND-1000 UV-spectrophotometer (NanoDrop Technologies).

Transcriptome in IFN-γ^+ (eYFP$^+$) CD4$^+$ T-cells

Naïve CD4$^+$ T-cells from dn*Rara*-IFN-γ^{eYFP} or littermate control IFN-γ^{eYFP} reporter mice were cultured under Th1 conditions. On day 7 of culture, following restimulation with PMA and ionomycin, eYFP$^+$ cells were sorted and total RNA was extracted for transcriptional profiling using Affymetrix Mouse Gene 2.0 ST arrays. Pre-processing and statistical analysis of gene expression data were done using Partek Genomics Suite 6.6. CEL files

were imported and expression intensities were summarised, normalised and transformed using Robust Multiarray Average algorithm. Two additional samples from eYFP$^+$ dn*Rara* or wild-type cells sorted without prior restimulation were included in the normalisation. These samples were not included in the analysis of differentially expressed genes. P values <0.05 and fold change in expression ≥ 1.5 or ≤ -1.5 were considered significant.

Transcriptome in Th1 differentiated cells

Sorted naïve CD4$^+$ T-cells from dn*Rara* or WT mice were polarised under Th1 conditions. On day 6 of culture cells were harvested and total RNA was extracted for microarray study or ChIP. RNA isolation, microarray and data processing performed by Miltenyi Biotec. Transcriptome analysis was performed using Agilent Whole Mouse Genome Oligo Microarrays 8X60K in accordance with manufacturer's protocol. Data analysis was performed using R/bioconductor and software packages therein (http://www.R-project.org ; http://www.bioconductor.org) or MS-Office Excel (Microsoft Inc.). Background corrected intensity values were normalized between arrays using quantile normalization. Quality controls include comparison of intensity profiles and a global correlation analysis. Differentially expressed genes were identified by statistical group comparisons on normalized (background corrected and quantile normalized) log2 transformed fluorescence intensities using Student´s t-test (two-tailed, equal variance). Reporters showing a p-value ≤ 0.05 and a median fold-change in expression ≥ 1.5 or ≤ -1.5 were considered as reliable candidates for altered gene expression. In addition, at least two of the replicate samples in the group with higher expression were required to have detection p-values ≤ 0.01.

Supplemental References

Feng, J., Liu, T. and Zhang, Y. (2011) Using MACS to Identify Peaks from ChIP-Seq Data. Current Protocols in Bioinformatics. 34:2.14:2.14.1–2.14.14.

Mucida, D., Park, Y., Kim, G., Turovskaya, O., Scott, I., Kronenberg, M., Cheroutre, H. (2007) Reciprocal TH17 and Regulatory T Cell Differentiation Mediated by Retinoic Acid. Science *317*, 256-260

Yong Zhang, Tao Liu1, Clifford A Meyer, Jérôme Eeckhoute, David S Johnson, Bradley E Bernstein, Chad Nusbaum, Richard M Myers, Myles Brown, Wei Li7 and X Shirley Liu. (2008) Model-based Analysis of ChIP-Seq (MACS). Genome Biology. 9:R 137
